# Supplementary material for: Oral health and oral health-related quality of life in patients with chronic peripheral facial nerve palsy with synkineses—A case-control-study
Source: PLoS One. 2022 Nov 17;17(11):e0276152. doi: 10.1371/journal.pone.0276152 (PMC9671450; doi:10.1371/journal.pone.0276152)
Supplement: S1 Checklist — (DOCX) [file pone.0276152.s001.docx]

**STROBE checklist:**

|  | Item No. | Recommendation | Page  No. |
| --- | --- | --- | --- |
| **Title and abstract** | 1 | (*a*) Indicate the study’s design with a commonly used term in the title or the abstract | 1 -2 |
|  |  | (*b*) Provide in the abstract an informative and balanced summary of what was done and what was found | 2 |
| Introduction | | | |
| Background/rationale | 2 | Explain the scientific background and rationale for the investigation being reported | 3 |
| Objectives | 3 | State specific objectives, including any prespecified hypotheses | 3-4 |
| Methods | | | |
| Study design | 4 | Present key elements of study design early in the paper | 4 |
| Setting | 5 | Describe the setting, locations, and relevant dates, including periods of recruitment, exposure, follow-up, and data collection | 4 |
| Participants | 6 | *Case-control study*—Give the eligibility criteria, and the sources and methods of case ascertainment and control selection. Give the rationale for the choice of cases and controls | 4-5 |
|  |  | *Case-control study*—For matched studies, give matching criteria and the number of controls per case | 4-5 |
| Variables | 7 | Clearly define all outcomes, exposures, predictors, potential confounders, and effect modifiers. Give diagnostic criteria, if applicable | 5-6 |
| Data sources/ measurement | 8* | For each variable of interest, give sources of data and details of methods of assessment (measurement). Describe comparability of assessment methods if there is more than one group | *Case group:*  *5- 6*  *Control group:*  *5-6* |
| Bias | 9 | Describe any efforts to address potential sources of bias | 6-7, 21-22 |
| Study size | 10 | Explain how the study size was arrived at | 4 |

| Quantitative variables | 11 | Explain how quantitative variables were handled in the analyses. If applicable, describe which groupings were chosen and why | 6 |  |
| --- | --- | --- | --- | --- |
| Statistical methods | 12 | (*a*) Describe all statistical methods, including those used to control for confounding | 6 |  |
|  |  | (*b*) Describe any methods used to examine subgroups and interactions | 6 |  |
|  |  | (*c*) Explain how missing data were addressed | not applicable, because all patients who wanted to participate and entered the study completed it by the end |  |
|  |  | *Case-control study*—If applicable, explain how matching of cases and controls was addressed | 6 |  |
|  |  | (*e*) Describe any sensitivity analyses | 6 |  |
| Results | | |  |  |
| Participants | 13* | (a) Report numbers of individuals at each stage of study—eg numbers potentially eligible, examined for eligibility, confirmed eligible, included in the study, completing follow-up, and analysed | *Case group:* 7  *Control group:* 7 | |
|  |  | (b) Give reasons for non-participation at each stage | not applicable, because all patients who wanted to participate and entered the study completed it by the end | |
| Descriptive data | 14* | (a) Give characteristics of study participants (eg demographic, clinical, social) and information on exposures and potential confounders | *Case group:* 7  *Control group:* 7 | |
|  |  | (b) Indicate number of participants with missing data for each variable of interest | not applicable | |
| Outcome data | 15* | *Case-control study—*Report numbers in each exposure category, or summary measures of exposure | 7-16 | |
|  |  | (*a*) Give unadjusted estimates and, if applicable, confounder-adjusted estimates and their precision (eg, 95% confidence interval). Make clear which confounders were adjusted for and why they were included | not applicable | |
| Main results | 16 | (*b*) Report category boundaries when continuous variables were categorized | 7-11 | |
|  |  | (*c*) If relevant, consider translating estimates of relative risk into absolute risk for a meaningful time period | not applicable | |
| Other analyses | 17 | Report other analyses done—eg analyses of subgroups and interactions, and sensitivity analyses | 11-16 | |
| Discussion | 18 | Summarise key results with reference to study objectives | | |
| Key results | 19 | Discuss limitations of the study, taking into account sources of potential bias or imprecision. Discuss both direction and magnitude of any potential bias | 17-23 | |
| Limitations | 20 | Give a cautious overall interpretation of results considering objectives, limitations, multiplicity of analyses, results from similar studies, and other relevant evidence | 21-21 | |
| Interpretation | 21 |  | 17-21 | |
| Generalisability |  | Discuss the generalisability (external validity) of the study results | 22 | |
| Other information | *22* | Give the source of funding and the role of the funders for the present study and, if applicable, for the original study on which the present article is based | No funding | |
| Funding | 23 |  | No funding | |
