# Supplementary material for: Oral health and oral health-related quality of life in patients with chronic peripheral facial nerve palsy with synkineses—A case-control-study
Source: PLoS One. 2022 Nov 17;17(11):e0276152. doi: 10.1371/journal.pone.0276152 (PMC9671450; doi:10.1371/journal.pone.0276152)
Supplement: S2 File — We developed a flyer with helpful tips and advice for patients with chronic facial palsy. The file is in Word format and can be modified accordingly. The flyer can be given to the patients after diagnosis in order to maintain the oral health of the affected patients in the long term. (DOCX) [file pone.0276152.s004.docx]

| 1. **Mundpflegehilfsmittel nicht vermeiden**   Versuchen Sie nicht, Mundspülungen zu vermeiden, trotz des höheren Flüssigkeitsverlustes durch den paretischen Mundwinkel. Wenn Flüssigkeit austritt, können Sie Ihre Lippen mit Hilfe Ihrer Finger schließen. Das hilft bei der ausreichenden Entfernung von Plaque und schützt Ihre Mundgesundheit. Üben Sie auch die Verwendung von Zahnseide.   1. **Paretische Seite nicht schonen**   Versuchen Sie im Alltag, Ihre paretische Seite beim Essen genauso zu benutzen wie Ihre nicht-paretische Seite. Eine übermäßige Beanspruchung der nicht-paretischen Seite kann zu starkem Verschleiß führen. Außerdem hat ein einseitiges Kauverhalten negative Auswirkungen auf das Kiefergelenk. Um weiterhin gleichmäßig kauen zu können, ist es wichtig, langsam und in kleinen Bissen zu essen.  **9 Nasendilatator**  Wenn Sie aufgrund Ihrer Gesichtsnervenlähmung zunehmend unter Mundtrockenheit leiden, können Sie nachts einen Nasendilatator tragen, um die Mundatmung zu minimieren und so die Mundtrockenheit zu verringern und die intensive Nasenatmung zu maximieren.  **10 Regelmäßige Zahnarztbesuche und professionelle Zahnreinigungen**  Nehmen Sie regelmäßige Zahnarztbesuche war. So erfahren Sie eine intensive prophylaktische Zahnpflege, gezielte Mundhygieneinstruktionen und ein individuelles Mundhygienetraining. Zu Beginn der Parese empfiehlt es sich, häufiger eine professionelle Zahnreinigung durchführen zu lassen, bis Sie die häusliche Mundpflege trotz Parese besser durchführen können. | **FAZIT**  **Es ist wichtig, dass Sie neben den primär eventuell wichtiger erscheinenden Folgen der Fazialisparese, wie Einschränkungen im Bereich der Augen, auch Ihre Mundgesundheit nicht vernachlässigen. Die Mundgesundheit ist für die Aufrechterhaltung der Allgemeingesundheit ebenso von großer Bedeutung.**  **Mit den genannten Tipps können Sie eine Verschlechterung Ihrer Mundgesundheit trotz Fazialisparese vorbeugen und zudem Ihre mundgesundheitsbezogene Lebensqualität langfristig aufrecht erhalten!**  *Ein Flyer von Lisa Strobelt, Anna-Maria Kuttenreich, Gerd Fabian Volk, Thomas Lehmann, Carien Beurskens, Catriona Neville und Ina Manuela Schüler*  *Kontakt:* 🖂 [*lisa.strobelt@gmx.de*](mailto:lisa.strobelt@gmx.de)  ✆ *036419329378*  Klinik und Poliklinik für Hals-, Nasen- und Ohrenheilkunde | 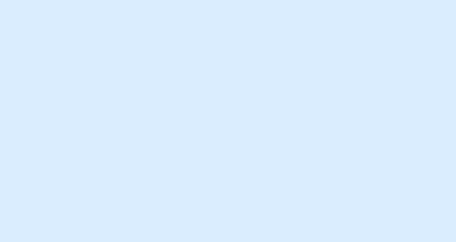  **Trotz Fazialisparese eine gute Mundgesundheit!**  **-**  **Hilfreiche Tipps für die tägliche Mundpflege** |
| --- | --- | --- |

| Liebe(r) PatientInnen,  aufgrund der teilweisen oder vollständigen Unbeweglichkeit Ihrer paretischen Gesichtshälfte können sich auch innerhalb Ihrer Mundhöhle Erkrankungen entwickeln.  Grund dafür ist, dass durch die eingeschränkte Funktion und Beweglichkeit Ihrer Gesichtsmuskulatur vermehrt Speisereste nach der Nahrungsaufnahme an Ihren Zahnflächen verbleiben, da diese nicht durch natürliche Selbstreinigungsmechanismen wie gezielte Muskelbewegungen der Wange entfernt werden können.  Das hat zur Folge, dass die oralen Beläge länger an Ihren Zahnflächen verbleiben und die Zahnsubstanz sowie Ihr Zahnfleisch angreifen, was wiederum zu einem erhöhten Auftreten von Karies und Parodontalerkrankungen führen kann.  Zur Vorbeugung erhalten Sie im folgenden Flyer 10 hilfreiche Tipps und Übungen, die Sie beim täglichen Zähneputzen integrieren und somit schnell und einfach Ihre orale Gesundheit trotz der Fazialisparese langfristig aufrecht erhalten können.  Lisa Strobelt | **Tipps für das richtige Zähneputzen und die langfristige Aufrechterhaltung der Mundgesundheit bei chronischer Fazialisparese**   1. **Wangendehnung und Massetermassage**   Nehmen Sie vor dem Zähneputzen den Daumen der nicht betroffenen Seite in den Mund und legen Sie die Finger dieser Hand auf die Außenseite der Wange (gegenüber dem Daumen) und drücken Sie dann mit dem Daumen die Wange nach außen. Somit erweitert sich der Raum zwischen Ihren Zahnreihen und der paretischen Wange und Sie gelangen während des Zähneputzens besser an die äußeren Zahnflächen zur besseren Plaqueentfernung auch in den hinteren Bereichen der Mundhöhle.   1. **Wangen aufblasen**   Einen ähnlichen Effekt hat das Aufblasen Ihrer Wangen vor dem Zähneputzen. Somit schaffen Sie mehr Raum und gelangen auch an hintere Zahnflächen mit größerem Erfolg.   1. **Vermeiden Sie „Clinching“/Klammern**   Wenn Sie das Gefühl haben, dass die Zähne und Backenzähne des Ober- und des Unterkiefers oft zusammenstehen, liegen Sie im Clinch. Dies kann die Spannung in den ohnehin schon steifen Wangen erhöhen. Es ist sehr wichtig, dass zwischen den Zähnen des Ober- und Unterkiefers immer ein wenig Platz ist, außer beim Essen und Schlucken. Spüren Sie, dass der Unterkiefer durch sein eigenes Gewicht entspannt ist. Versuchen Sie, Ihren Unterkiefer im normalen Alltag zu entspannen; machen Sie es sich leichter, indem Sie auch zwischen den Lippen ein wenig Platz lassen.  Klinik und Poliklinik für Hals-, Nasen- und Ohrenheilkunde | Konzentrieren Sie sich auf ein entspanntes Gesicht, zunächst mit sanft geschlossenen Lippen. Dann entspannen Sie den Kiefer, lassen Sie ihn "schwer" sein, so dass sich die Lippen langsam öffnen und der Mund durch sein eigenes Gewicht auffällt. Schließen Sie dann langsam den Mund wieder und lassen Sie die Lippen sanft aneinander liegen Dann entspannen Sie den Kiefer, wobei die Lippen entspannt aufeinander liegen. Wiederholen Sie dies einige Male.   1. **Spezialisierten Fazialistherapeut ansprechen**   Um die Muskelübungen richtig auszuüben und zu intensivieren empfiehlt es sich, parallel zum Muskeltraining daheim eine gezielte physiotherapeutische/logopädische Behandlung in Anspruch zu nehmen.   1. **Eigenen Sie sich einen systematischen Putzablauf an**   Beginnen Sie mit dem Putzen auf der paretischen Seite beispielsweise zuerst im Oberkiefer, dann im Unterkiefer. Anschließend widmen Sie sich Ihrer nicht paretischen Seite. Somit erfährt Ihre paretische Seite zum Anfang des Zähneputzens Ihre volle Konzentration, da diese Seite aufgrund der eingeschränkten Muskelbewegung mehr Unterstützung bei der Entfernung oraler Beläge benötigt. Die adäquate Reinigung Ihrer nicht paretischen Seite darf aber ebenso nicht vernachlässigt werden!   1. **Intraorale Massage mit der elektrischen Zahnbürste**   Zu empfehlen ist die Verwendung einer elektrischen Zahnbürste. Diese reinigt die Zahnflächen effektiver und kann vor allem auch in den Zahnzwischenräumen mehr Beläge entfernen. Außerdem können Sie Ihre elektrische Zahnbürste zum Zwecke der intraoralen Massage Ihrer Wangenmuskulatur gleichzeitig umfunktionieren. Fahren Sie einfach mit dem Bürstenkopf von innen über Ihre Wange und erfahren somit einen lohnenswerten Massageeffekt.  Klinik und Poliklinik für Hals-, Nasen- und Ohrenheilkunde |
| --- | --- | --- |

| **8 Do not spare the paretic side**  In everyday life, try to use your paretic side just as much as your non-paretic side when eating. Excessive strain on the non-paretic side can lead to severe wear and tear. In addition, one-sided chewing behaviour has negative effects on the jaw joint. To continue chewing evenly it is important to eat slowly with small bites.  **9 Nasal dilator**  If you suffer increasingly from dry mouth due to your facial nerve palsy, you can wear a nasal dilator at night to minimise mouth breathing and thus decrease dry mouth and maximise intentional nasal breathing.  **10 regular visits to the dentist**  Make regular visits to the dentist.  You will receive intensive prophylactic dental care, specific oral hygiene instructions and individual oral hygiene training. At the beginning of the paresis, it is recommended that you have your teeth professionally cleaned more often until you are better able to perform oral care at home despite the paresis.  Klinik und Poliklinik für Hals-, Nasen- und Ohrenheilkunde | **CONCLUSION**  **It is important that you do not neglect your oral health in addition to the consequences of facial nerve palsy, which may appear to be primarily more important, such as limitations in the area of the eyes. Oral health is equally important for maintaining general health.**  **With the tips mentioned above, you can prevent a deterioration of your oral health despite facial paresis and also maintain your oral health-related quality of life in the long term!**  *A flyer by Lisa Strobelt, Anna-Maria Kuttenreich, Gerd Fabian Volk, Thomas Lehmann, Carien Beurskens, Catriona Neville and Ina Manuela Schüler*  *contact:* 🖂 [*lisa.strobelt@gmx.de*](mailto:lisa.strobelt@gmx.de)  ✆ *036419329378*  Klinik und Poliklinik für Hals-, Nasen- und Ohrenheilkunde | 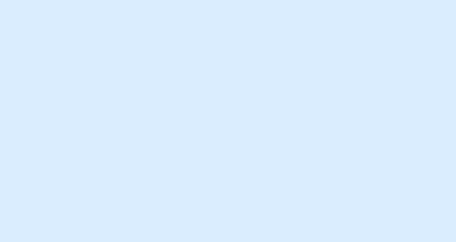  **Good oral health despite facial nerve palsy!**  **-**  **Helpful tips for daily oral care** |
| --- | --- | --- |

| Dear patient(s),  Due to the partial or complete immobility of the paretic half of your face, diseases can also develop within your oral cavity.  The reason for this is that due to the limited function and mobility of your facial muscles, more food residues remain on the surfaces of your teeth after eating, as these cannot be removed by natural self-cleaning mechanisms such as targeted muscle movements of the cheek.  As a result, oral plaque stays on your tooth surfaces longer and attacks the tooth structure and your gums, which in turn can lead to an increased incidence of caries and periodontal disease.  For prevention, the following flyer provides you with 10 helpful tips and exercises that you can integrate into your daily tooth brushing and thus quickly and easily maintain your oral health in the long term despite facial nerve palsy.  Lisa Strobelt | **Tips for proper tooth brushing and long-term maintenance of oral health in**  **chronic facial nerve palsy**   1. **Cheek stretching and masseter massage**   Before brushing your teeth, place the thump of your non-affected side in your mouth and the fingers of that hand on the outside of the cheek (opposite of the thump).Than press with the thump the cheek outwards. This expands the space between your teeth and the paretic cheek and gives you more access to the outer surfaces of your teeth for better plaque removal, even in the back of the mouth.   1. **Inflate cheeks**   Inflating your cheeks before brushing has a similar effect. This creates more space and allows you to reach the back of your teeth with greater success.     1. **Avoid clinching**   If you feel that the teeth and molars of upper and lower jaw are often together you are clinching. This can give more tension in the already stiff cheek. It is very important that, except when eating and swallowing, there is always a little space between the teeth of the upper and lower jaw. Feel that the lower jaw is relaxed by his own weight. Try to relax your lower jaw in your normal daily life; make it easier with also a little space between your lips. Concentrate on a relaxed face, first with the lips gently closed. Then relax the jaw, let it be ‘heavy’ so that the lips slowly part and the mouth falls open by its own weight. Next, slowly close the mouth again and let the lips rest gently against one another. Then relax the jaw, with the lips together in a relaxed way. Repeat this a number of times.  Klinik und Poliklinik für Hals-, Nasen- und Ohrenheilkunde | 1. **Consult a specialized facial therapist**   In order to practice and intensify the muscle exercises correctly, it is advisable to look for a specific physiotherapeutic/logopedic treatment parallel to the muscle training at home.   1. **Adopt a systematic brushing routine**   Start brushing on the paretic side, for example, first in the upper jaw, then in the lower jaw. Then turn your attention to the non-paretic side. This way, your paretic side receives your full concentration at the beginning of brushing, as this side needs more support in removing oral plaque due to the restricted muscle movement. However, adequate cleaning of your non-paretic side must not be neglected either!   1. **Intraoral massage with the electric toothbrush**   It is recommended to use an electric toothbrush. It cleans the tooth surfaces more effectively and can remove more plaque, especially in the interdental spaces. You can also use your electric toothbrush for an intraoral massage of your cheek muscles at the same time. Simply run the brush head over your cheek from the inside for a rewarding massage effect.   1. **Do not avoid oral care aids**   Try not to avoid mouth rinses, despite the higher fluid loss through the paretic corner of your mouth. When liquid is leaking you can close your lips with help of your fingers. This will assist in adequate plaque removal and protect your oral health. Also practice flossing.  Klinik und Poliklinik für Hals-, Nasen- und Ohrenheilkunde |
| --- | --- | --- |
