# Supplementary material for: Oral health and oral health-related quality of life in patients with chronic peripheral facial nerve palsy with synkineses—A case-control-study
Source: PLoS One. 2022 Nov 17;17(11):e0276152. doi: 10.1371/journal.pone.0276152 (PMC9671450; doi:10.1371/journal.pone.0276152)
Supplement: S3 File — (DOCX) [file pone.0276152.s005.docx]

**Studienprotokoll**

Mundgesundheit und mundgesundheitsbezogene Lebensqualität von Patienten mit Fazialisparese am Universitätsklinikum Jena

Kontrollierte Querschnittstudie

| Studienakronym: | Mundgesundheit und mundgesundheitsbezogene Lebensqualität |
| --- | --- |
| Protokollversion: | Version vom 10.08.2020 |

# Studienprotokoll

Mundgesundheit und mundgesundheitsbezogene Lebensqualität von Patienten mit Fazialisparese am Universitätsklinikum Jena

Bei Personen- und Funktionsbezeichnungen im folgenden Protokoll wird der leichteren Lesbarkeit halber die männliche Sprachform verwendet und meint, wenn nicht anders hervorgehoben, stets auch die weibliche sowie diverse.

Inhalt

[Studienprotokoll 2](#_Toc46335447)

[Abkürzungsverzeichnis 4](#_Toc46335448)

[1. Allgemeine Informationen 5](#_Toc46335449)

[1.1 Beteiligte Personen und Institutionen 5](#_Toc46335450)

[1.2 Zusammenfassung 6](#_Toc46335451)

[1.3 Synopse 7](#_Toc46335452)

[2. Hintergrund 8](#_Toc46335453)

[2.1 Ausgangssituation 8](#_Toc46335454)

[2.2 Fragestellung und Begründung des Vorhabens 9](#_Toc46335455)

[3. Ziele der Studie 11](#_Toc46335456)

[3.1 Primäres Ziel 11](#_Toc46335457)

[3.2 Sekundäre Ziele 12](#_Toc46335458)

[4.Studiendesign und –beschreibung 13](#_Toc46335459)

[4.1 Art der Studie 13](#_Toc46335460)

[4.2 Art der Therapiezuordnung 13](#_Toc46335461)

[4.3 Art der Vergleichsgruppe 13](#_Toc46335462)

[4.4 Umfang der Studie 13](#_Toc46335463)

[4.5 Patienten- und Probandenrekrutierung 13](#_Toc46335464)

[4.6 Zeitplan 14](#_Toc46335465)

[5. Auswahl der Versuchspersonen 14](#_Toc46335466)

[5.1 Einschlusskriterien 14](#_Toc46335467)

[5.2 Ausschlusskriterien für die Auswahl der Versuchspersonen 15](#_Toc46335468)

[6. Ablauf der Studie 15](#_Toc46335469)

[6.1 Aufklärung und Einwilligung 15](#_Toc46335470)

[6.2 Fragebogen 16](#_Toc46335471)

[6.3 Orale Untersuchung 16](#_Toc46335472)

[6.4 OHIP-G14-Fragebogen 17](#_Toc46335473)

[6.5 Dokumentation 17](#_Toc46335474)

[6.6 Analyse der gewonnenen Daten 18](#_Toc46335475)

[6.7 Vergleich der Ergebnisse zwischen Patienten und Probanden sowie von Patienten mit unterschiedlichen Formen und unterschiedlicher Erkrankungsdauer 18](#_Toc46335476)

[6.8 Tabellarischer Ablaufplan 18](#_Toc46335477)

[7. Biometrie und Fallzahlplanung 18](#_Toc46335478)

[8. Datenmanagement 19](#_Toc46335479)

[8.1 Patienten- und Probandenidentifikationsliste 19](#_Toc46335480)

[8.2 Datenerhebung/Dokumentationsbögen 19](#_Toc46335481)

[8.3 Aufbewahrung der Studienunterlagen 20](#_Toc46335482)

[9. Ethische Erwägungen 20](#_Toc46335483)

[10. Finanzierung 20](#_Toc46335484)

[11. Literatur 20](#_Toc46335485)

[12. Anlagen 22](#_Toc46335486)

# Abkürzungsverzeichnis

| API | Approximalraum-Plaque-Index |
| --- | --- |
| max. ST | Maximale Sondierungstiefe |
| mod. SBI | Modifizierter Sulkus-Blutungsindex |
| OHIP-G14 | Oral Health Impact Profile-Germany 14 |
| PBI | Papillenblutungsindex |
| PSI | Parodontaler Screening Index |
| TI | Turesky-Plaque-Index |

# 1. Allgemeine Informationen

## 1.1 Beteiligte Personen und Institutionen

**Studienleiter**

Priv.-Doz. Dr. med. Gerd Fabian Volk

Klinik und Poliklinik für Hals-, Nasen- und Ohrenheilkunde

Universitätsklinikum Jena

Am Klinikum 1

07747 Jena

03641-9329396

E-Mail: [fabian.volk@med.uni-jena.de](mailto:fabian.volk@med.uni-jena.de)

**Weitere Studienleiterin**

Priv.-Doz. Dr. med. dent. Ina Manuela Schüler

Poliklinik für Kieferorthopädie, Sektion Präventive Zahnheilkunde und Kinderzahnheilkunde

Zentrum für Zahn-, Mund- und Kieferheilkunde Jena, Universitätsklinikum Jena

An der Alten Post 4

07743 Jena

03641-9323721/ 03641-9329729

E-Mail: [ina.schueler@med.uni-jena.de](mailto:ina.schueler@med.uni-jena.de)

**Beteiligte Wissenschaftler**

Untersucherin:

cand. med. dent. Lisa Strobelt

Klinik und Poliklinik für Hals-, Nasen- und Ohrenheilkunde

Poliklinik für Kieferorthopädie, Sektion Präventive Zahnheilkunde und Kinderzahnheilkunde

Zentrum für Zahn-, Mund- und Kieferheilkunde Jena, Universitätsklinikum Jena

0175/2276748

E-Mail: [lisa.strobelt@uni-jena.de](mailto:lisa.strobelt@uni-jena.de)

Wissenschaftliche Mitarbeiterin des FNZ Jena:

M. Sc. Anna-Maria Kuttenreich

Klinik und Poliklinik für Hals-, Nasen- und Ohrenheilkunde

06341-9329398

E-Mail: [anna-maria.kuttenreich@med.uni-jena.de](mailto:anna-maria.kuttenreich@med.uni-jena.de)

Biometrikerin:

M.A Elisabeth Settke

Institut für Medizinische Statistik, Informatik und Datenwissenschaften

03641-9-396952

E-Mail: [elisabeth.settke@med.uni-jena.de](mailto:elisabeth.settke@med.uni-jena.de)

**Beteiligte Einrichtungen**

HNO-Klinik, Fazialis-Nerv-Zentrum, Universitätsklinikum Jena

Zahnklinik Jena, Universitätsklinikum Jena

## 1.2 Zusammenfassung

Eine Fazialisparese kann zur Unbeweglichkeit der betroffenen Gesichtshälfte führen (Trepel, 2017), sodass davon auszugehen ist, dass Speisereste an Zahnflächen nicht durch natürliche Selbstreinigungsmechanismen wie gezielte Muskelbewegungen oder Speichel, die in der Literatur (Elferich & Tittmann, 2004) beschrieben sind, entfernt werden können. Neben der Nahrungsaufnahme ist bei den Patienten die Mundpflege beeinträchtigt (Jakobsen & Sticher, 2015). Durch eine Verschlechterung der Mundhygiene wird das Risiko für orale Infektionen erhöht und folglich auch das sonstiger Erkrankungen (Hellwege, 2018).

Ziel dieser Studie ist es, die Mundgesundheit und mundgesundheitsbezogene Lebensqualität von Patienten mit verschiedenen Formen der Fazialisparese sowie die Mundgesundheit in Abhängigkeit zu Variablen der Fazialisparese wie z.B. der Erkrankungsdauer und –ursache, die im Rahmen der Routine des Fazialis-Nerv-Zentrums erhoben werden, zu untersuchen.

In einer kontrollierten Querschnittstudie sollen Mundgesundheitsparameter und die mundgesundheitsbezogene Lebensqualität zu einem Zeitpunkt untersucht werden. Da Patienten in unterschiedlichen zeitlichen Stadien (akute Phase, chronische Phase) dokumentiert werden sollen, wird das Setting der HNO-Klinik Jena gewählt. Dort werden sowohl Patienten mit akuten Paresen als auch Patienten mit chronischen Formen der Fazialisparese behandelt. Die Mundgesundheitsparameter und die mundgesundheitsbezogene Lebensqualität dieser Patientengruppe sollen mit einer Kontrollgruppe verglichen werden. Dazu sollen Probanden ohne eine jemals von einem Arzt diagnostizierte Fazialisparese nach Alter und Geschlecht mit den entsprechenden Patienten gematcht werden.

Als primäres Kriterium für die Beurteilung der Mundgesundheit soll die Einschätzung der parodontalen Gesundheit dienen, die mit Hilfe des parodontalen Screening-Indexes (Hellwege, 2018; Weber, 2017) von einer Zahnmedizinerin analysiert wird. Mit weiteren etablierten Indizes^[[1]](#footnote-1)^ werden zudem der Karies- und Plaquebefall, die Blutungsneigung der Gingiva, Auffälligkeiten der Mundschleimhaut sowie das Vorhandensein von Halitosis erfasst und zwischen den Gruppen verglichen. Die mundgesundheitsbezogene Lebensqualität wird mit Hilfe des validierten Oral Health Impact Profile-Germany-14-Fragebogen (John, 2002; Slade 1997) erfasst.

Angenommen wird eine Fallzahl von 60 Patienten und 60 Probanden.

Beide Versuchsgruppen werden aus der Ambulanzebene und Stationsebene der HNO-Klinik des Universitätsklinikums Jena rekrutiert. Zusätzlich können weitere Probanden zur besseren Vergleichbarkeit aus dem Tinnitus-Zentrum der HNO-Klinik Jena sowie aus in Thüringen niedergelassenen Zahnarztpraxen rekrutiert werden.

## 1.3 Synopse

| **Titel der Studie** | Mundgesundheit und mundgesundheitsbezogene Lebensqualität von Patienten mit Fazialisparese am Universitätsklinikum Jena |
| --- | --- |
| **Kurzbezeichnung der Studie (Akronym)** | Mundgesundheit und mundgesundheitsbezogene Lebensqualität |
| **Studienleiter** | Priv.-Doz. Dr. Gerd Fabian Volk |
| **Stellvertreterin des Studienleiters** | Priv.-Doz. Dr. med. dent. Ina Manuela Schüler |
| **Untersucherin** | cand. med. dent Lisa Strobelt |
| **Indikation/Zielpopulation/Erkrankung** | Probanden: gesunde Probanden ohne jemals von einem Arzt diagnostizierte Fazialisparese  Patienten: Patienten mit unterschiedlichen Formen der Fazialisparese in unterschiedlichen zeitlichen Stadien/ mit unterschiedlicher Erkrankungsdauer |
| **Studiendesign/Methodik** | Kontrollierte Querschnittstudie |
| **Ziele der klinischen Prüfung/Zielstellung** | Primäres Ziel:  Erfassen der Mundgesundheit und mundgesundheitsbezogenen Lebensqualität von Patienten und Probanden:   - Erfassen des Zahnstatus   (Lockerungsgrades, bestehende Versorgungen)   - Erfassen der parodontalen Gesundheit   (PSI, Vorhandensein von Zahnstein, Attachementverlust und maximale Sondierungstiefe)   - Erfassen der Blutungsneigung der Gingiva - Erfassen des Kariesbefall   (PUFA-Index und Kariesaktivität)   - Erfassen des Plaquebefalls   (Turesky-Plaque-Index und API)   - Erfassen von Mundschleimhautauffälligkeiten - Erfassen des Vorhandenseins von Halitosis   Sekundäre Ziele:   - Erfassen der mundgesundheitsbezogenen Lebensqualität |
| **Zielgrößen/-kriterien/ Endpunkte** | Primäre Zielgröße:  PSI (Parameter für chronische Parodontitis)  Max. ST (maximale Sondierungstiefe)  mod. SBI (Parameter für Blutungsneigung)  PBI (Parameter zur Erfassung der Quantität der Papillenblutung)  PUFA (Parameter für odontogene Infektionen infolge unbehandelter Karies)  API (Parameter für approximalen / interdentalen Plaquebefall)  TI (Parameter für Plaquebefall)  Sekundäre Zielgrößen:  Mundgesundheitsbezogene Lebensqualität |
| **Patientenzahl** | 60 Probanden und 60 Patienten  Gesamtzahl der Versuchspersonen: n=120 |
| **Einschlusskriterien** | Probanden: keine Vorgeschichte einer Fazialisparese, entsprechend Alter und Geschlecht mit Patienten gematcht  Patienten: Patienten mit allen Formen der Fazialisparese (von einem Arzt diagnostiziert) |
| **Ausschlusskriterien** | Multimorbide Patienten/Probanden  Fehlende Einwilligung  Zahnlose Patienten/Probanden  Patienten/Probanden mit folgenden diagnostizierten Grunderkrankungen:   - Hämophilie A und B - Epilepsie - Aids |
| **Behandlungen/Verfahren, Behandlungsplan (inkl. Nachsorge)** | Für die Probanden und Patienten zu einem Zeitpunkt:   - Aufklärung und Einwilligung in die Studie - Ausfüllen eines ersten Fragebogens (Fragebogen 1, subjektive Beurteilung der eigenen Mundhygiene und Mundgesundheit) - Mundgesundheitsuntersuchung - Ausfüllen eines zweiten Fragebogens (OHIP-G14-Fragebogen zur mundgesundheitsbezogenen Lebensqualität) - Keine Behandlung der Patienten und keine Nachsorge im Rahmen der Studie, sondern im Rahmen der individuell wahrzunehmenden jährlichen zahnärztlichen Kontrolluntersuchung |
| **Zeitplan (Studiendauer)** | Beginn: 01.05.2020  Ende: 30.12.2023  Patientenbezogen:  Dauer der studienbedingten Untersuchung:  60 Minuten |
| **Prüfzentren** | n = 1 |
| **Statistische Methoden** | Deskriptive Statistik und Interferenzstatistik |
| **Finanzierung** | Hausmittel |

#

# 2. Hintergrund

## Ausgangssituation

Bei der Lähmung des Nervus facialis wird in Abhängigkeit von der Lokalisation der Schädigung zwischen peripherer und zentraler Parese unterschieden (Kaschke, Behrbohm & Nawka, 2009). Das Hauptsymptom beider Formen der Fazialisparese ist die schlaffe Lähmung der Gesichtsmuskulatur (Trepel, 2017). Dabei ist sowohl bei einer supranukleären Schädigung, die zur zentralen Parese führt, als auch bei einer infranukleären Schädigung, die eine periphere Parese zur Folge hat, die periorale Region mit betroffen (Jakobsen & Sticher, 2015). Im Bereich der Wange fehlt die Muskelspannung im Musculus buccinator oder sie ist zu hoch, woraus Bissverletzungen im Wangenbereich der Patienten resultieren können. Zudem besteht die Gefahr, dass bei fehlender Aktivität und Sensibilität in der Wange nach der Nahrungsaufnahme Speisereste in der Buccinatortasche verbleiben, wodurch u.a. die Entstehung von Entzündungen der Schleimhaut begünstigt wird (Jakobsen & Sticher, 2015). Im Bereich der Lippenmuskulatur kommt es infolge der schlaffen Lähmung der Gesichtsmuskulatur zu einem herabhängenden Mundwinkel auf der betroffenen Seite und einem unvollständigen Lippenschluss. Beispielsweise beim Trinken können die Patienten die Flüssigkeit deshalb nicht im Mund halten (Trepel, 2017). Eine Schädigung des Nervus facialis im Canalis nervi facialis vor dem Abgang der Chorda tympani führt möglicherweise zusätzlich zu einer Mundtrockenheit (Trepel, 2017). Vor allem der Speichel sorgt aber neben gezielten Muskelbewegungen für einen natürlichen Selbstreinigungsmechanismus der Mundhöhle, wenn an Zahnflächen Nahrungsreste verbleiben (Elferich & Tittmann, 2004). Ist zu wenig Speichel vorhanden, sodass eine geringere Speichelflussrate resultiert, kommt es zur Erhöhung des Kariesrisikos. Starke Xerostomie kann zur Austrocknung der Mundschleimhaut führen. Folglich kommt es zu Einrissen, Läsionen und Schmerzen (Elsäßer & Ludwig, 2017). Eine Xerostomie kann somit auf längere Zeit zu einer deutlichen Einschränkung der Lebensqualität führen (Zenk, Leins & Bozzato, 2005).

Durch die Veränderungen im Gesichtsbereich und vor allem durch die Einschränkungen in der Beweglichkeit der perioralen Region ist die Nahrungsaufnahme, aber auch die Mundpflege bei den betroffenen Patienten beeinträchtigt (Jakobsen & Sticher, 2015). Wenn ein unvollständiger Lippenschluss besteht und die Patienten Schwierigkeiten haben, Flüssigkeiten im Mund zu halten, dann lässt sich vermuten, dass die Betroffenen auch Probleme beim Ausspülen des Mundes nach der Zahnreinigung, beim Ausspucken von Zahnpasta oder bei der Anwendung von Mundspüllösungen haben. Wenn die Mundpflege von Patienten erschwert ist, dann stellt sich die Frage, ob dies auch die resultierende Mundhygiene und die damit verbundene Mundgesundheit bei den Patienten verschlechtert. Eine 2013 veröffentlichte Studie gibt einen ersten Hinweis darauf, dass eine periphere Gesichtslähmung die Mundhygiene beeinträchtigt, was in der Folge zu Munderkrankungen führen kann (Kato et al., 2013).

## Fragestellung und Begründung des Vorhabens

Aufgrund der geringen Evidenz bleibt bislang die Frage offen, wie sich die Situation der Mundhygiene bei Patienten mit Fazialisparese genau darstellt, ob sich durch die Erkrankung und die erschwerten Bedingungen der Mundpflege Defizite in der Mundgesundheit erkennbar machen, ob diese in Korrelation mit der Erkrankungsdauer stehen, ob Unterschiede der Mundgesundheit zwischen der paretischen und nicht paretischen Gesichtshälfte der Patienten verzeichnet werden können und ob sich Differenzen zu nicht erkrankten Personen aufdecken lassen.

Eine mangelnde Mundhygiene spiegelt sich vorerst durch Zahnerkrankungen wie Karies und Erkrankungen des Zahnhalteapparates wieder. Foeter ex ore (Mundgeruch), eine erhöhte Blutungsneigung der Gingiva, Zahnfleischschwellungen und Schmerzen stellen die Folgen dar (Elsäßer & Ludwig, 2017). Im weiteren Verlauf wird durch die Verschlechterung der Mundhygiene und die damit auftretenden Schmerzen in der Mundhöhle die Nahrungsaufnahme erschwert, denn nur mit einer gesunden Mundhöhle ist diese bestmöglich umsetzbar (Elferich & Tittmann, 2004). Aber nicht nur aufgrund letzterer Tatsachen stellt eine Verschlechterung der Mundhygiene ein großes Risiko für die betroffenen Patienten dar. Die Bundeszahnärztekammer verdeutlichte bereits 2002 auf ihrer Pressekonferenz in Berlin mit dem Slogan *„Gesunde Zähne – gesunder Körper*“ die Wechselwirkungen zwischen bakteriellen Erkrankungen des Parodonts und Herz-Kreislauf-Erkrankungen sowie Erkrankungen der Gefäße (Elferich & Tittmann, 2004). In untersuchten Präparaten von entsprechenden Gefäßwänden gelang der Nachweis von parodontalpathogenen Erregern. Es wurde gezeigt, dass bei Parodontitis auftretende Mikroorganismen auch in Verbindung mit Arteriosklerose stehen (Mastragelopulos, et al., 2004). Parodontalpathogene Mikroorganismen können zur Aktivierung der Thrombozytenaggregation führen, die Produktion von Entzündungsmolekülen begünstigen sowie die Bildung von Schaumzellen aus Makrophagen ermöglichen, was zu einer Ansammlung von arteriosklerotischen Plaque in den Gefäßwänden führt (Jacek, 2018). Das Bindegewebe des Gingivasaumes verfügt über ein dichtes, sehr permeables subsulkuläres Gefäßnetz (Hellwege, 2018). Bei Zerstörung des Parodonts können Bakterien so über die entstandenen Zahnfleischtaschen in den Blutkreislauf gelangen und dort auch die systemische Gesundheit der Patienten gefährden (Jacek, 2018). Aber nicht nur über die Gingiva ist eine Ausbreitung von Mikroorganismen in den gesamten Körper möglich. Stille Aspiration, die mit einem erhöhten Risiko für Lungenentzündungen verbunden ist, sowie rissige Schleimhaut gelten als weitere mögliche Ausbreitungswege (Elsäßer & Ludwig, 2017). Es wird deutlich, dass orale Infektionen Ausgangspunkt für die Entstehung weiterer Krankheiten sein können (Hellwege, 2018). Man nimmt neben der bereits beschriebenen Wechselwirkung zu Arteriosklerose u.a. auch Verbindungen zu den Erkrankungen Endokarditis, koronare Herzkrankheit und Schlaganfall an. Auch eine Korrelation mit dem Insulinbedarf von Diabetikern und Komplikationen in der Schwangerschaft wird vermutet (Hellwege, 2018). Festzustellen ist, dass mithilfe einer besseren Zahngesundheit auch die Allgemeingesundheit profitieren kann. Das Zitat von Klaus-Dieter Hellwege aus dem 2018 im Thieme Verlag erschienen Buch *die Praxis der zahnmedizinischen Prophylaxe* bringt dies auf den Punkt: „Mit sauberen und gesunden Zähnen lässt es sich nicht nur besser, sondern auch länger leben.“ ( Zitat, Klaus-Dieter Hellwege, 2018, Kapitel 3, S.47).

Darüber hinaus leistet eine gute Mundhygiene sowohl einen positiven als auch ausschlaggebenden Beitrag zum Wohlbefinden und der Lebensqualität (Elsäßer & Ludwig, 2017).

Aus den dargelegten Überlegungen heraus lässt sich folgende Hypothese formulieren:

Wenn eine gute Mundhygiene durch eine erschwerte Mundpflege nicht mehr erreicht werden kann, dann führt dies zu einem Unwohlsein der Patienten.

Begünstigt durch die gehemmten Möglichkeiten der Mundpflege, Nahrungsaufnahme und Flüssigkeitszufuhr distanzieren sich die betroffenen Patienten aus ihrer sozialen Umgebung (Jakobsen & Sticher, 2015).

Zusammenfassend wird einerseits deutlich, dass eine gute Mundhygiene und Mundgesundheit eine große Relevanz für eine hohe Lebensqualität, die Partizipation im Alltag sowie eine gesunde systemische und psychische Aufstellung hat. Andererseits kann festgestellt werden, dass eine Verschlechterung der Mundhygiene weitreichende Folgen für die Gesundheit birgt und eine solche Situation zeitnah erkannt und behandelt werden sollte. Besonders in Prädilektionsstellen wie den Fissuren kann sich aktiver Biofilm ablegen und bei unregelmäßiger und verzögerter Entfernung zu Demineralisation an der Zahnoberfläche führen. Die entstehenden Kariesläsionen sind dann irreversibel (Roulet, Fath, & Zimmer, 2017).

Aufgrund der genannten Hinweise darauf, dass bei Patienten mit Fazialisparese die Mundhygiene und die damit verbundende Mundgesundheit durch eine nur erschwert mögliche Mundpflege sowie die Einschränkung der Selbstreinigungsmechanismen beeinträchtigt sein könnte, soll ein Überblick über die Mundgesundheit der Patienten mit Fazialisparese am Universitätsklinikum Jena geschaffen werden. Falls mögliche Defizite und Unterschiede zu nicht Erkrankten aufgedeckt werden können, ist es möglich, eine zahnärztliche Therapiebedürftigkeit abzuleiten, zukünftig gezielt auf diese Patientengruppe einzugehen und deren Mundhygiene und die damit verbundene allgemeine Gesundheit zu verbessern.

# 3. Ziele der Studie

## 3.1 Primäres Ziel

Primäres Ziel der Studie ist das Untersuchen und Erfassen der Mundgesundheit von Patienten mit unterschiedlichen Formen der Fazialisparese sowie das Dokumentieren der Mundgesundheit dieser Patientengruppe in Abhängigkeit zu Variablen der Fazialisparese wie z.B. der Erkrankungsdauer und –ursache, die im Rahmen der Routine des Fazialis-Nerv-Zentrums erhoben werden. Ein besonderes Augenmerk soll dabei auf den parodontalen Gesundheitszustand der Patienten gelegt werden. Zur Erfassung der Mundgesundheit soll ein kompletter Zahnstatus inklusive vorhandene Versorgungen und Lockerungen der Zähne erhoben werden. Zur Erfassung der parodontalen Gesundheit sowie der Gesundheit der Gingiva soll der PSI (Parameter für eine chronische Parodontalerkrankung, entwickelt von Deutscher Gesellschaft für Parodontologie 2002, Vgl. Hellwege 2018), das Vorhandensein von Zahnstein, die maximalen Sondierungstiefen der Zahnfleischtaschen (in mm), der Attachementverlust (in mm), der modifizierte SBI nach Lange et al. 1986 (Parameter für das Auftreten einer Sulkusblutung, Vgl. Weber 2017) sowie der PBI nach Saxer und Mühlemann 1975 (Parameter zur Bestimmung der Quantität der Papillenblutung, Vgl. Hellwege 2018) von einer Zahnmedizinerin bestimmt werden. Als weitere Mundgesundheitsparameter sollen der Karies- und Plaquebefall dokumentiert werden. Dabei soll der Kariesbefall mittels des PUFA-Index nach Monse et al. 2010 (Parameter für odontogene Infektionen infolge unbehandelter Karies, Vgl. Kühnisch & Heinrich-Weltzien, 2020) und der Erfassung der Kariesaktivität bestimmt werden. Der Plaquebefall soll mithilfe des Turesky-Plaque-Index (Turesky et al. 1970, Parameter für Plaquebefall, Modifizierung des Plaqueindexes nach Quigley und Hein 1962, Vgl. Peter Gängler et. al 2005) sowie dem API nach Lange et al. 1986 (Parameter für approximalen/interdentalen Plaquebefall, Vgl. Weber 2017) untersucht werden. Außerdem soll die Mundschleimhaut gründlich inspiziert und das Vorhandensein von Halitosis mittels der organoleptischen Diagnostik (Vgl. Weber 2010) beurteilt werden.

Dadurch wird ein umfangreicher Überblick über die orale Situation bei Patienten mit Fazialisparese geschaffen. Die einzelnen Mundgesundheitsparameter sollen dabei mit einer Kontrollgruppe, die hinsichtlich derselben Messverfahren untersucht wird, verglichen werden.

## 3.2 Sekundäre Ziele

Als sekundäre Ziele sollen zum einen die mundgesundheitsbezogene Lebensqualität der Patienten mit Hilfe des validierten OHIP-G14-Fragebogens^[[2]](#footnote-2)^ festgestellt werden, um auch auf die psycho-soziale Komponente der Erkrankung eingehen zu können. Auch die Ergebnisse der Fragebögen sollen sowohl mit den Variablen der Fazialisparese wie z.B. der Erkrankungsdauer und –ursache, die im Rahmen der Routine des Fazialis-Nerv-Zentrums erhoben werden, als auch mit den Werten einer Kontrollgruppe, verglichen werden.

# 4.Studiendesign und -beschreibung

## 4.1 Art der Studie

Bei der geplanten Studie handelt es sich um eine kontrollierte Querschnittstudie.

## 4.2 Art der Therapiezuordnung

Es werden bei den Patienten keine Therapien im Sinne einer studienbedingten Intervention durchgeführt. Routinemäßig ausgeführte zahnärztliche Untersuchungen erfolgen indikationsgerecht.

Auf Wunsch können die Patienten während oder nach der Untersuchung fachkundige Ratschläge für eine optimale Mundpflege von der Zahnmedizinerin erhalten und offene Fragen klären.

## 4.3 Art der Vergleichsgruppe

Eine entsprechende Kontrollgruppe aus nicht an Fazialisparese erkrankten und freiwillig teilnehmenden Probanden wird genau wie die Patientengruppe aus dem Setting der Ambulanzebene sowie Stationsebene der HNO-Klinik des Universitätsklinikums Jena rekrutiert.

In dem Fall, dass aus diesem Setting zu wenige Probanden für eine gute Vergleichbarkeit rekrutiert werden können, erfolgen studienbezogene Untersuchungen an entsprechenden Probanden aus dem Tinnitus-Zentrum der HNO-Klinik des Universitätsklinikums Jena oder an Patienten aus in Thüringen niedergelassenen Zahnarztpraxen.

Es wird dabei nach Alter und Geschlecht gematcht.

## 4.4 Umfang der Studie

Insgesamt werden 60 Patienten und 60 Probanden eingeschlossen. Die genaue Fallzahlplanung erfolgte mithilfe des Institutes für medizinische Statistik, Informatik und Datenwissenschaften in Jena unter Verwendung des Programms G*power^[[3]](#footnote-3)^.

## 4.5 Patienten- und Probandenrekrutierung

Beide Kollektive werden von der Untersucherin rekrutiert und untersucht.

Die Patienten werden dabei aus der HNO-Klinik des Universitätsklinikum Jenas im Rahmen der dienstags stattfindenden EMG-Sprechstunde, im Rahmen des teilstationären Fazialis-Parese-Trainings des Fazialis-Nerv-Zentrums Jenas sowie die Patienten mit akuten Fazialisparesen innerhalb des stationären Aufenthalts rekrutiert.

Die Rekrutierung geeigneter Probanden erfolgt ebenfalls in der HNO-Klinik des Universitätsklinikums Jena. Es werden Probanden einbezogen, die sich ebenfalls in stationärer Behandlung befinden, die aber nicht auf eine Fazialisparese zurückzuführen ist. Geeignete Probanden dürfen auch in ihrer Vergangenheit keine Fazialisparese erlitten haben. Des Weiteren wird versucht, Patienten über das Tinnitus-Zentrum am Universitätsklinikum Jena zu rekrutieren. Darüber hinaus können Probanden auch aus in Thüringen niedergelassenen Zahnarztpraxen angeworben werden, falls dies für ein besseres Vergleichsergebnis zur Patientengruppe nötig wird.

## 4.6 Zeitplan

Erwartete Gesamtstudiendauer: 44 Monate

Meilensteine im Zeitverlauf (Monat/Jahr):

| Vorbereitungen/Planung | 05/2020 |
| --- | --- |
| Einschluss erster Patient | 08/2020 |
| Einschluss letzter Patient | 05/2021 |
| Einschluss erster Proband | 08/2020 |
| Einschluss letzter Proband | 07/2021 |
| Ende statistische Auswertung | 12/2022 |
| Abschlussbericht | 12/2023 |

# 5. Auswahl der Versuchspersonen

## 5.1 Einschlusskriterien

**Patienten n=60**

Von einem Arzt diagnostizierte Fazialisparese

Alle Altersgruppen

Schriftliche Einwilligung des Patienten oder des Erziehungsberechtigten (bei Patienten unter 18 Jahren) zur Studienteilnahme

bezahnt

nicht an einer der folgenden diagnostizierten Grunderkrankung leidend:

Hämophilie A und B

Epilepsie

Aids

**Probanden n=60**

Auswahl hinsichtlich des Alters und Geschlechts als Kontrollgruppe zu zuvor untersuchten Patienten

Keine jemals diagnostizierte Fazialisparese

Schriftliche Einwilligung des Probanden oder des Erziehungsberechtigten (bei Probanden unter 18 Jahren) zur Studienteilnahme

bezahnt

nicht an einer der folgenden diagnostizierten Grunderkrankung leidend:

Hämophilie A und B

Epilepsie

Aids

## 5.2 Ausschlusskriterien

**Patienten**

Multimorbide Patienten

Keine diagnostizierte Fazialisparese

Fehlende Einwilligung des Patienten oder des Erziehungsberechtigten (bei Patienten unter 18 Jahren) zur Studienteilnahme

zahnlos

an einer der folgenden diagnostizierten Grunderkrankung leidend:

Hämophilie A und B

Epilepsie

Aids

**Probanden**

Multimorbide Probanden

hinsichtlich des Alters und Geschlechts nicht matchbar mit zuvor untersuchten Patienten

diagnostizierte Fazialisparese aktuell oder in Vergangenheit

fehlende Einwilligung des Probanden oder des Erziehungsberechtigten (bei Probanden unter 18 Jahren) zur Studienteilnahme

zahnlos

an einer der folgenden diagnostizierten Grunderkrankung leidend:

Hämophilie A und B

Epilepsie

Aids

# 6. Ablauf der Studie

### 6.1 Aufklärung und Einwilligung

Am Anfang der Studie wird der Versuchsperson (Patient oder Proband) ein Aufklärungsbogen (siehe Anhang), der über die Art der Studie, die Ziele, den Ablauf, die Dauer, die Vorteile, mögliche Risiken, die Datenauswertung, den Datenschutz und die Teilnehmerrechte informiert, sowie eine Einverständniserklärung (siehe Anhang) ausgehändigt. Nach gründlichem Lesen beider Formulare überzeugt sich die Untersucherin davon, dass die Aufklärung vom Patient/Proband verstanden wurde und gibt ihm die Möglichkeit, offene Fragen anzusprechen und diese ausführlich beantwortet zu bekommen. Der Versuchsperson wird anschließend ausreichend Zeit gegeben, sich über die Studienteilnahme zu entscheiden. Im nächsten Schritt wird der Teilnehmer dann gebeten, bei Interesse an der Studie, seine Kenntnisnahme sowie sein Einverständnis eigenhändig schriftlich zu dokumentieren. Diese Einwilligung bezieht sich ausdrücklich auch auf die Erhebung und Verarbeitung von personenbezogenen Daten. Aufgrund dessen werden die Patienten/Probanden ausführlich und explizit über Zweck und Umfang der Erhebung und Verwendung dieser Daten informiert.

Der Patient/Proband erhält im Anschluss eine Kopie der unterschriebenen Einwilligungserklärung, das Original verbleibt bei der Untersucherin. Der Teilnehmer kann jederzeit ohne Angaben von Gründen und ohne Nachteil für seine weitere Behandlung diese Einwilligung zurückziehen, die Untersuchung abbrechen und aus der Studie austreten. Der Zeitpunkt des Teilnahmeabbruches wird in diesem Fall dokumentiert. Bei fehlender Unterzeichnung der Einwilligung wird die Versuchsperson nicht in die Studie eingeschlossen.

Erst wenn die Person beide Formulare bejaht und unterzeichnet hat, der Person eine entsprechende Kopie der Unterlagen ausgehändigt wurde und alle sonstigen Fragen geklärt sind, wird mit der Untersuchung begonnen.

### 6.2 Fragebogen

Vor der Untersuchung der Versuchspersonen wird den Teilnehmern ein Fragebogen (Fragebogen 1, subjektive Bewertung der eigenen Mundhygiene und Mundgesundheit, siehe Anhang) ausgehändigt und mit dem Patient/Proband in einem gemeinsamen Interview besprochen. Im Falle, dass der Patient/ Proband den Bogen lieber allein ausfüllen möchte, wird ihm dies ermöglicht. Die Patienten /Probanden werden gebeten, den Fragebogen so wahrheitsgemäß und intuitiv wie möglich auszufüllen.

Durch den Fragebogen werden wichtige Informationen wie zum Beispiel die Einstellung der Person zur eigenen Mundhygiene, die bisherige Durchführung der Zahnreinigung sowie mögliche Risikofaktoren für die Entwicklung einer minderwertigeren Mundhygiene erfasst, um festzustellen, ob auftretende Defizite der Mundhygiene auch bereits vor dem Zustand der Fazialisparese bestanden oder ob bei der Versuchsperson eventuell auch genetisch oder motorisch bedingte Einschränkungen bestehen, wodurch eine schlechtere Mundhygiene nicht mehr unmittelbar auf die Fazialisparese zurück zu führen wäre. Der Fragebogen soll somit für die Untersucherin die Möglichkeit bieten, den Patient/Proband hinsichtlich seiner Mundhygiene besser kennenzulernen und ihn in Bezug dessen erfolgreicher einschätzen zu können.

### 6.3 Orale Untersuchung

Die orale Untersuchung erfolgt bei den Patienten nach demselben Schema wie bei den Probanden.

Während der Mundhygieneuntersuchung inspiziert die Untersucherin die gut ausgeleuchtete Mundhöhle und diagnostiziert die Zähne, die Gingiva sowie die Mundschleimhaut sowie vorhandenen Zahnersatz als orale Strukturen. Dabei wird der komplette Zahnstatus der Person inklusive Versorgungen der Zähne sowie deren Lockerungsgrad, die parodontale Gesundheit mittels des PSI, dem Vorhandensein von Zahnstein, der maximalen Sondierungstiefe sowie dem Attachementverlust, die Blutungsneigung des Zahnfleisches mittels des modifizierten SBI nach Lange et. al 1986 sowie dem PBI nach Mühlemann und Saxer 1975, der Kariesbefall mittels dem PUFA-Index nach Monse et al. 2010 und der Kariesaktivität, der Plaquebefall mittels dem API nach Lange et al. 1986 und dem Turesky-Plaque-Index nach Turesky et al. 1970, Auffälligkeiten an der Mundschleimhaut nach WHO-Einteilung sowie das Vorhandensein von Halitosis untersucht und dokumentiert. Zur Erhebung und Dokumentation des Zahnstatus sowie der verschiedenen Mundgesundheitsparameter dient ein papierbasierter Befundbogen (siehe Anhang), der im Anschluss in ein Excel- Dokument überführt wird.

Nach der Befundaufnahme werden intraorale Fotoaufnahmen angefertigt.

Auf Wunsch können die Patienten der Untersucherin offene Fragen bezüglich der Mundpflege stellen und fachkundige Ratschläge zur Durchführung einer optimalen Zahnreinigung sowie Biofilmmanagement erhalten.

Die Durchführung der Untersuchungen erfolgt dabei in den Räumlichkeiten des Universitätsklinikums Jena, insbesondere in der Ambulanzebene für Hals-, Nasen und Ohrenheilkunde. Eine Untersuchung der Probanden ist darüber hinaus auch in einer niedergelassenen zahnärztlichen Praxis denkbar. Die teilnehmenden Patienten und Probanden werden bezüglich ihrer Erkrankung unabhängig von der geplanten Studie in der HNO-Klinik des Universitätsklinikums Jena standardisiert untersucht und therapiert. Die ermittelten Daten über die Mundgesundheitssituation der Patienten/Probanden werden diesen mitgeteilt und vertraulich behandelt, nehmen aber auf die weitere zahnärztliche sowie hno-ärztliche Behandlung der Patienten keinen Einfluss.

### 6.4 OHIP-G14-Fragebogen

Nach der Mundhygieneuntersuchung bekommt die Person einen weiteren Fragebogen ausgehändigt, der die empfundene mundgesundheitsbezogene Lebensqualität wiederspiegeln soll. Dazu wird der validierte OHIP-G14-Fragebogen verwendet. Die Versuchsperson wird gebeten, auch diesen wahrheitsgemäß und intuitiv auszufüllen. Alternativ wird der Fragebogen im gemeinsamen Interview besprochen.

### 6.5 Dokumentation

Es erfolgt eine pseudonymisierte Datenerfassung mithilfe einer Patienten-/Probandenidentifikationsliste (siehe Anhang), einem manuell auszufüllenden Befundbogen (siehe Anhang) sowie mithilfe von zwei manuell auszufüllenden Fragebögen (siehe Anhang). Der papierbasierte Befundbogen wird in ein digitales Excel-Formular überführt.

### 6.6 Analyse der gewonnenen Daten

Die gewonnenen Daten werden hinsichtlich der einzelnen Mundgesundheitsparameter für jeden Patient/ Proband ausgewertet. Dabei erfolgt eine Auswertung der Parameter bei den Patienten auch getrennt für die paretische und nicht paretische Seite sowie bei den Probanden für die linke und rechte Mundhälfte, um im Anschluss einen Seitenvergleich anstellen zu können.

Hinsichtlich der gewonnenen Daten der Fragebögen erfolgt eine Auswertung für jede gestellte Frage einzeln.

Zudem werden die gewonnenen Daten nach Alter, Geschlecht, Form, Dauer und Ursache der Fazialisparese ausgewertet.

### 6.7 Vergleich der Ergebnisse

Die gewonnenen und ausgewerteten Daten werden zunächst zwischen den Patienten und im Anschluss mit denen der Kontrollgruppe verglichen.

Verglichen werden hinsichtlich der Mundgesundheitsparameter:

- Pro Patient:
- paretische und nicht paretische Mundhälfte
- Pro Proband:
- rechte und linke Mundhälfte
- zwischen den Patienten:
- beide Mundhälften gesamt
- paretische Mundhälfte
- nicht paretische Mundhälfte
- Zwischen den Patienten und Probanden:
- beide Mundhälften gesamt
- paretische Mundhälfte mit entsprechender Mundhälfte des Probanden
- nicht paretische Mundhälfte mit entsprechender Mundhälfte des Probanden

Verglichen werden hinsichtlich der Daten aus den Fragebögen:

- Die Antworten jeder Frage zwischen den Patienten
- Die Antworten jeder Frage zwischen den Probanden
- Die Antworten jeder Frage zwischen den Patienten und den Probanden

### 6.8 Tabellarischer Ablaufplan

| 1. | Aufklärung über die Studie und Einholen der Einverständniserklärung |
| --- | --- |
| 2. | Aushändigung, Beantwortung und Dokumentation des 1. Fragebogens  (Fragebogen 1, subjektive Beurteilung der eigenen Mundhygiene und Mundgesundheit) |
| 3. | Datenerhebung im Rahmen der zahnärztlichen Mundhygieneuntersuchung |
| 4. | Dokumentation der gewonnenen Daten |
| 5 | Aushändigung, Beantwortung und Dokumentation des 2. Fragebogens  (OHIP-G14-Fragebogen zur mundgesundheitsbezogenen Lebensqualität) |
| 6. | Analyse und Auswertung der Daten |
| 7. | Vergleich der Ergebnisse |
| 8. | Publikation der Ergebnisse |

# Biometrie und Fallzahlplanung

Eine ähnliche Studie, die belegt, dass eine periphere Gesichtslähmung die Nahrungsaufnahme beeinträchtigt und somit die Mundhygiene verschlechtert, was zu Munderkrankungen führen kann, wurde von 2009 bis 2011 in der HNO- Klinik Nabon University Itabashi Hospital und Nihon University Dental Hospital in Japan durchgeführt. Diese Studie beinhaltete 30 Patienten sowie 30 Kontrollpersonen, woran sich die geplante Studie orientiert.

Die notwendige Anzahl von Versuchspersonen wurde mit Hilfe des Institutes für Medizinische Statistik, Informatik und Datenwissenschaften Jena ermittelt. Es wurde das Programm G*power verwendet. Bei der Berechnung der genauen Fallzahl wurden folgende Werte angenommen:

- Wilcoxon-Mann-Whitney- Test (two groups)
- Tail: two
- parent distribution: min ARE
- effect size d: 0,6666667
- $\alpha$ err prop: 0,05
- Power (1-$\beta$ err prop): 0,8
- allocation ratio N1/N2: 1

Somit wurde eine Fallzahl von 43 Patienten und 43 Probanden berechnet. Das macht eine Gesamtzahl von insgesamt 86 Versuchspersonen. Zur Berücksichtigung von Drop-out-Fällen wird von einer Gesamtzahl von 120 Versuchspersonen (jeweils 60 Patienten/Probanden pro Gruppe) ausgegangen.

# Datenmanagement

## Patienten- und Probandenidentifikationsliste

Es erfolgt eine pseudonymisierte Erfassung der Patienten-/Probandendaten. Für jeden Patienten/Proband wir ein individuelles Pseudonym verwendet, durch welches allein die Identität der Versuchsperson nicht erkannt werden kann. Es wird eine Patienten-/Probandenidentifikationsliste geführt. In dieser wird die Patienten-/Probandenidentifikationsnummer mit dem vollständigen Namen des Teilnehmers, dessen Geburtsdatum, dessen Alter, dessen Beruf sowie bei den Patienten mit deren Form, Schwere, Dauer und Ursache der Fazialisparese in Verbindung gebracht, um eine spätere Identifikation der Versuchspersonen zu ermöglichen. Dieses Dokument wird absolut vertraulich aufbewahrt und gelangt nicht in die Hände Dritter. Es wird mindestens 10 Jahre archiviert.

## Datenerhebung/Dokumentationsbögen

Die für die Studie notwendige Datenerhebung erfolgt in der HNO-Klinik des Universitätsklinikums Jena und gegebenenfalls in niedergelassenen Zahnarztpraxen in Thüringen. Die Daten werden primär in Form von papierbasierten Dokumentationsbögen erfasst und anschließend in entsprechende digitale Excel-Tabellen überführt.

## Aufbewahrung der Studienunterlagen

Alle studienbegleitenden Unterlagen werden in Originalform für mindestens 10 Jahre beim Untersucher nach Abschluss der Studie aufbewahrt. Die Dokumente werden vertraulich an einem sicheren Ort aufbewahrt. Dabei wird darauf geachtet, dass die Dokumentationsunterlagen nicht gemeinsam mit der Identifikationsliste der Patienten/Probanden gelagert werden.

# Ethische Erwägungen

Bei der Planung der Studie sind die Grundsätze der „Deklaration von Helsinki“ (Stand Oktober 2013) berücksichtigt. Es sind keine negativen Auswirkungen auf die Patienten und Probanden aufgrund der Studie zu erwarten. Für die Patienten und Probanden besteht kein Risiko. Die Behandlung der Patienten wird durch die Studie nicht verändert. Für die Versuchspersonen ergibt sich ein durch die Studie bedingter einmaliger Zeitaufwand von ca. 60 Minuten.

Die Empfehlungen der „Guten Klinischen Praxis“, gültig seit dem 17.1.1997, werden, sofern zutreffend, berücksichtigt.

# Finanzierung

Es ist keine Entschädigung für die Teilnahme vorgesehen. Die Finanzierung der Studie erfolgt aus Haushaltsmitteln. Es gibt keine anderen Sponsoren oder Interessenskonflikte.

# Literatur

Elferich, B. & Tittmann, D. (2004). *Die Therapie des Facio-Oralen Ttrakts: F.O.T.T. nach kay Coombes.* (R. Nusser-Müller-Busch, Hrsg.) Berlin; Heidelberg; New York: Springer. S.78-79

Elsäßer, G. & Ludwig, E. (2017). Handbuch der Mundhygiene. In Bundeszahnärztekammer (Hrsg.). Berlin.

Gängler, P., Hoffmann, T., Schwenzer, N. & Willershausen, B. (2005)

*Konservierende Zahnheilkunde und Parodontologie.* Michael Ehrenfeld(Hrsg.), Zweite Aufl.

Stuttgart: Georg-Thieme-Verlag KG, S. 252

Hellwege, K.-D. (2018). *Die Praxis der Zahnmedizinischen Prophylaxe, Ein Leitfaden für die Individualprophylaxe für Zahnärzte und Mitarbeiter.* Stuttgart: Georg Thieme verlag KG.S. 35, S.46-47, S.88-90

Jacek, R. (November 2018). Parodontitis ist ein Risiko für den gesamten Körper. *ZMK aktuell* , S. 788-790.

Jakobsen, D. & Sticher, H. (2015). Die Therapie des Facio-Oralen Trakts: F.O.T.T. nach Kay Coombes. In R. Nusser-Müller-Busch (Hrsg.). Berlin, Deutschland: Springer.S.160-161,S.168, S.171

John, M. T. (2002). The German version of the Oral Health Impact Profile – Translation and psychometric properties. *European Journal of Oral Science* , S. 110,425-433.

Kaschke, O., Behrbohm, H. & Nawka, T. (2009). *Kurzlehrbuch Hals-Nasen-Ohren-Heilkunde.* Stuttgart: Georg Thieme Verlag KG.S.67

Kato, Y., Kamo, H., Kobayashi, A., Abe, S., Okada-Ogawa, A., Noma, N. et al. (Juni 2013). Quantitative evaluation of Oral Function in Acute and Recovery Phase of Idiopathic Facial Palsy; A Preliminary Controlles Study. *Clinical Otolaryngology* , S. 231-236.

Kühnisch, J. & Heinrich-Weltzien, R. (2020). *Kinderzahnmedizin.* (J. Kühnisch, Hrsg.) Berlin, Deutschland: Quintessenz-Verlags-GmbH.

Mastragelopulos, N., Rogge, S., Kielbassa, A., Haraszthy, V., Zambon, J., Brunkwall, J. et al. (April 2004). Parodontitis und Arteriosklerose. *Gefässchirurgie* .

Roulet, J.-F., Fath, S. & Zimmer, S. (. (2017). *Zahnmedizinische Prophylaxe: Lehrbuch und Praxisleitfaden.* Deutschland: Elsevier, Urban&Fischer.S.58, S.60, S.68

Slade, G. D. (1997). Derivation and validation of a short‐form oral health impact profile. *Community Dentistry and Oral Epidemiology* , S. 25(4), 284-290.

Trepel, M. (2017). *Neuroanatomie, Struktur und Funktion.* Deutschland: Elsevier, Urban&Fischer.S.68, S.122, S.148

Weber, T. (2010). *Memorix Zahnmedizin.* Stuttgart, New York: Georg-Thieme-Verlag KG. S.109

Weber, T. (2017). *Memorix Zahnmedizin*. Stuttgart, New York: Georg-Thieme-Verlag KG. S.118-126

Zenk, J., Leins, P. & Bozzato, A. (2005). *HNO Praxis heute, Funktionsstörungen und funktionelle Störungen.* (E. Biesinger, & H. Iro, Hrsg.) Heidelberg: Springer. S.2

# Anlagen

Aufklärungsbogen Patientengruppe

Aufklärungsbogen Kontrollgruppe

Einwilligungserklärung Patienten

Einwilligungserklärung Probanden

Identifikationsliste

Fragebogen 1

OHIP-G14-Fragebogen

Befundbogen

1. Die genaue Angabe folgt auf Seite 10. [↑](#footnote-ref-1)
2. Genaueres zum Fragebogen folgt bei Gliederungspunkt 6.4. [↑](#footnote-ref-2)
3. Genaue Angaben sind unter 7. Biometrie und Fallzahlplanung ersichtlich. [↑](#footnote-ref-3)
