# Supplementary material for: Oral health and oral health-related quality of life in patients with chronic peripheral facial nerve palsy with synkineses—A case-control-study
Source: PLoS One. 2022 Nov 17;17(11):e0276152. doi: 10.1371/journal.pone.0276152 (PMC9671450; doi:10.1371/journal.pone.0276152)
Supplement: S4 File — (DOCX) [file pone.0276152.s006.docx]

**Study protocol**

Oral health and oral health-related quality of life of patients with facial nerve palsy at Jena University Hospital

controlled cross-sectional study

| study acronym: | Oral health and oral health-related quality of life |
| --- | --- |
| protocol version: | version of 10 august 2020 |

# Study protocol

Oral health and oral health-related quality of life of patients with facial nerve palsy at Jena University Hospital

For ease of reading, masculine form is used for personal and functional designations in the following protocol and, unless otherwise emphasised, always also means feminine as well as diverse form.

# Table of contents

[Study protocol 2](#_Toc77190927)

[Table of contents 2](#_Toc77190928)

[Abbreviations 4](#_Toc77190929)

[1 General information 5](#_Toc77190930)

[1.1 Involved persons and institutions 5](#_Toc77190931)

[1.2 Summary 6](#_Toc77190932)

[1.3 Synopsis 7](#_Toc77190933)

[2 Background 8](#_Toc77190934)

[2.1 Initial situation 8](#_Toc77190935)

[2.2 Research question and justification 9](#_Toc77190936)

[3 Objectives 10](#_Toc77190937)

[3.1 Primary objective 10](#_Toc77190938)

[3.2 Secondary objective 11](#_Toc77190939)

[4. Study design and description 11](#_Toc77190940)

[4.1 Type of study 11](#_Toc77190941)

[4.2 Type of therapy assignment 11](#_Toc77190942)

[4.3 Type of comparison group 12](#_Toc77190943)

[4.4 Scope of the study 12](#_Toc77190944)

[4.5 Patient and subject recruitment 12](#_Toc77190945)

[4.6 Schedule 12](#_Toc77190946)

[5 Selection 13](#_Toc77190947)

[5.1 Inclusion criteria 13](#_Toc77190948)

[5.2 Exclusion criteria 13](#_Toc77190949)

[6 Procedure of the study 13](#_Toc77190950)

[6.1 Information and consent 13](#_Toc77190951)

[6.2 Questionnaire 14](#_Toc77190952)

[6.3 Oral examination 14](#_Toc77190953)

[6.4 OHIP-G14 questionnaire 15](#_Toc77190954)

[6.5 Documentation 15](#_Toc77190955)

[6.6 Data analysis 15](#_Toc77190956)

[6.7 Comparison of results 16](#_Toc77190957)

[6.8 Tabular schedule 16](#_Toc77190958)

[7 Biometrics and sample size planning 17](#_Toc77190959)

[8 Data management 17](#_Toc77190960)

[8.1 Identification list 17](#_Toc77190961)

[8.2 Data collection/documentation forms 17](#_Toc77190962)

[8.3 Storage of study documents 18](#_Toc77190963)

[9 Ethical considerations 18](#_Toc77190964)

[10 Funding 18](#_Toc77190965)

[11 References 18](#_Toc77190966)

[12 Attachments 19](#_Toc77190967)

# Abbreviations

| API | approximal plaque index |
| --- | --- |
| MPD | maximum probing depth |
| mod. SBI | modified sulcus bleeding index |
| OHIP-G14 | Oral Health Impact Profile-Germany 14 |
| PBI | papillary bleeding index |
| PSI | periodontal screening Index |
| TI | Turesky plaque index |

# 1 General information

## 1.1 Involved persons and institutions

**Director**

Priv.-Doz. Dr. med. Gerd Fabian Volk

Department of Otolaryngology and Facial-Nerve-Center Jena, Jena University Hospital

Am Klinikum 1

07747 Jena

Germany

03641-9329396

E-Mail: [fabian.volk@med.uni-jena.de](mailto:fabian.volk@med.uni-jena.de)

**Further study leader**

Priv.-Doz. Dr. med. dent. Ina Manuela Schüler

Section for Preventive Dentistry and Pediatric Dentistry at the Center for Dentistry, Oral and Maxillofacial Surgery of Jena University Hospital

An der Alten Post 4

07743 Jena

Germany

03641-9323721/ 03641-9329729

E-Mail: [ina.schueler@med.uni-jena.de](mailto:ina.schueler@med.uni-jena.de)

**Investigator**

cand. med. dent. Lisa Strobelt

Section for Preventive Dentistry and Pediatric Dentistry at the Center for Dentistry, Oral and Maxillofacial Surgery of Jena University Hospital

Department of Otolaryngology and Facial-Nerve-Center Jena, Jena University Hospital Germany

0175/2276748

E-Mail: [lisa.strobelt@uni-jena.de](mailto:lisa.strobelt@uni-jena.de)

**Research assistant at the FNZ Jena:**

M. Sc. Anna-Maria Kuttenreich

Department of Otolaryngology and Facial-Nerve-Center Jena, Jena University Hospital

03641-9329398

E-Mail: anna-maria.kuttenreich@med.uni-jena.de

**Biometrican:**

M.A Elisabeth Settke

Institute for Medical Statistics, Informatics and Data Science Jena

03641-9-396952

E-Mail: [elisabeth.settke@med.uni-jena.de](mailto:elisabeth.settke@med.uni-jena.de)

**Participating institutions**

Section for Preventive Dentistry and Pediatric Dentistry at the Center for Dentistry, Oral and Maxillofacial Surgery of Jena University Hospital

Department of Otolaryngology and Facial-Nerve-Center Jena, Jena University Hospital

## 1.2 Summary

Facial nerve palsy lead to immobility of the affected side of face (Trepel, 2017). Assumable, food residues on tooth surfaces cannot be removed by natural self-cleaning mechanisms such as targeted muscle movements or saliva, which are described in literature (Elferich & Tittmann, 2004). In addition to food intake, oral hygiene is impaired in patients (Jakobsen & Sticher, 2015). Deterioration of oral hygiene increases the risk of oral infections and consequently of other diseases (Hellwege, 2018).

Aim of this study is to investigate the oral health and oral health-related quality of life of patients with different forms of facial nerve palsy, as well as oral health in relation to facial nerve palsy variables such as disease duration and cause, which are collected as part of routine investigation at Facial Nerve Centre Jena.

In a controlled cross-sectional study, oral health parameters and oral health-related quality of life will be investigated at one time. Since patients in different temporal stages (acute phase, chronic phase) are to be documented, the setting of the Ears-, Nose- and Throat (ENT-) clinic Jena was chosen. There are both patients, with acute paresis and patients with chronic forms of facial nerve palsy. Oral health parameters and oral health-related quality of life of these patients groups should be compared with a control group. For this, controls without facial nerve palsy ever diagnosed by a doctor will be matched with corresponding patients according to age and gender.

Primary criterion for assessment of oral health will be the assessment of periodontal health, which will be analysed by a dentist using parodontal screening index (PSI; Hellwege, 2018; Weber, 2017). Other established indices are used to record caries and plaque incidence, bleeding tendency of gingiva, abnormalities of oral mucosa and presence of halitosis. These indices are compared between the two groups. Oral health-related quality of life will be assessed using the validated OHIP-G14- questionnaire (John, 2002; Slade 1997).

It is assumed a case number of 60 patients and 60 controls.

Both study groups will be recruited from ambulance level and ward level of ENT-clinic at Jena University Hospital. For better comparability, further controls can be recruited from the tinnitus centre of ENT-clinic Jena as well as from dental practices established in Thuringia.

## 1.3 Synopsis

| **Title of the study** | Oral health and oral health-related quality of life of patients with facial nerve palsy at Jena University Hospital |
| --- | --- |
| **Short name of the study (acronym)** | Oral health and oral health-related quality of life |
| **Head of study** | Priv.-Doz. Dr. Gerd Fabian Volk |
| **further head of study** | Priv.-Doz. Dr. med. dent. Ina Manuela Schüler |
| **Investigator** | cand. med. dent Lisa Strobelt |
| **Indication/target population/disease** | Controls: healthy persons without facial nerve palsy ever diagnosed by a doctor  Patients: patients with different forms of facial nerve palsy in different temporal stages/ with different disease duration |
| **Study design/methodology** | Controlled cross-sectional study |
| **Clinical trial objectives/Objectives** | Primary objective:  To record oral health and oral health-related quality of life in patients and controls:  - To record dental status  (tooth mobility, restorations)  - To assess periodontal health  (PSI, presence of calculus, attachment loss and MPD)  - Assessment of gingiva bleeding  - Assessment of caries  (PUFA index and caries activity)  - Assessment of plaque infestation  (TI and API)  - Detection of oral mucosal abnormalities  - Detection of halitosis  Secondary objective:  - To assess oral health-related quality of life |
| **Target variables/criteria/endpoints** | Primary target variable:  PSI (chronic periodontitis parameter).  MPD (maximum probing depth)  mod. SBI (parameter for bleeding tendency)  PBI (parameter for quantity of papillary bleeding)  PUFA (parameter for odontogenic infections due to untreated caries)  API (parameter for approximal / interdental plaque infestation)  TI (parameter for plaque infestation)  Secondary target variable:  Oral health-related quality of life |
| **Number of participants** | 60 patients an 60 controls |
| **Total number of participants** | n=120 |
| **Inclusion criteria** | Controls: no history of facial nerve palsy, matched with patients according to age and gender  Patients: patients with all forms of facial nerve palsy (diagnosed by a doctor) |
| **Exclusion criteria** | Multimorbid patients/controls  Lack of informed consent  Edentulous patients/controls  Patients/controls with following diagnosed diseases:  - Haemophilia A and B  - Epilepsy  - AIDS |
| **Treatments/procedures, treatment plan (incl. follow-up)** | For controls and patients at one time:  - Informed study consent  - Completion of an initial questionnaire (questionnaire 1, subjective assessment of own oral hygiene and oral health)  - Oral health examination  - Completion of a second questionnaire (OHIP-G14 questionnaire on oral health-related quality of life)  - No treatment of patients and no follow-up care within the study, but within the individually annual dental check-up |
| **Schedule (study duration)** | Start: 01.05.2020  End: 30.12.2023  Patient-related:  Duration of study-related examination:  60 minutes |
| **Study sites** | n = 1 |
| **Statistical methods** | Descriptive statistics and interference statistics |
| **Funding** | Home remedies |

# 2 Background

## 2.1 Initial situation

In facial nerve palsy, a distinction is made between peripheral and central paresis depending on the location of damage (Kaschke, Behrbohm & Nawka, 2009). Main symptom of both forms of facial nerve palsy is a flaccid paralysis of facial musculature (Trepel, 2017). Perioral region is affected in both supranuclear damage, which leads to central palsy, and infranuclear damage, which results in peripheral palsy (Jakobsen & Sticher, 2015). In the patient’s cheek region, muscle tension in the buccinator muscle is missing or too high, which can result in bite injuries in patient's cheek region. In addition, there is a risk that, in absence of cheek activity and sensitivity, food residues remain in the buccinator pocket after food intake, which, among other things, favours the development of inflammations of mucosa (Jakobsen & Sticher, 2015). In the area of lip musculature, flaccid paralysis of the facial musculature leads to a drooping corner of mouth on the affected side and incomplete lip closure. For example, when drinking, patients are therefore unable to hold the liquid in their mouth (Trepel, 2017). Damage to facial nerve in the facial nerve canal in front of tympanic chord may also lead to dry mouth (Trepel, 2017). However, saliva in particular, in addition to targeted muscle movements, provides a natural self-cleaning mechanism of oral cavity when food residues remain on tooth surfaces (Elferich & Tittmann, 2004). If there is too less saliva, resulting in a lower saliva flow rate, risk of caries increases. Severe xerostomia can lead to dehydration of oral mucosa. Consequently, tears, lesions and pain occur (Elsäßer & Ludwig, 2017). Xerostomia can lead to a significant reduction in quality of life over a longer period of time (Zenk, Leins & Bozzato, 2005).

Due to changes in facial area and especially due to restrictions in mobility of the perioral region, food intake, but also oral hygiene is impaired in affected patients (Jakobsen & Sticher, 2015). If there is incomplete lip closure and patients have difficulty keeping liquids in their mouths, it can be assumed that those affected also have problems rinsing their mouths after cleaning their teeth, spitting out toothpaste or using mouth rinses. If patients' oral care is more difficult, then the question arises whether this also worsens the resulting oral hygiene and associated oral health in patients. A study published in 2013 provides the first indication that peripheral facial nerve palsy impairs oral hygiene, which can subsequently lead to oral diseases (Kato et al., 2013).

## 2.2 Research question and justification

Due to the lack of evidence, the question remains as to what exactly oral hygiene looks like in patients with facial nerve palsy, whether deficits in oral health becomes apparent due to more difficult conditions of oral care, whether these correlate with the duration of the disease, whether differences in oral health between paretic and non-paretic side can be recorded and whether differences can be detected in comparison to non-diseased controls.

A lack of oral hygiene is initially reflected by dental diseases such as caries and diseases of periodontium. Foeter ex ore (bad breath), an increased bleeding tendency of gingiva, gingiva swelling and pain represent consequences (Elsäßer & Ludwig, 2017). In further course, deterioration of oral hygiene and resulting pain in oral cavity make it more difficult to eat, because only with a healthy oral cavity is it possible to do so in the best possible way (Elferich & Tittmann, 2004). But it is not only because of latter facts that a deterioration in oral hygiene represents a great risk for patients. As early as 2002 on a press conference in Berlin, the German Dental Association used the slogan "Healthy teeth - healthy body" to illustrate interactions between bacterial diseases of periodontium and cardiovascular diseases as well as diseases of the blood vessels (Elferich & Tittmann, 2004). In examined preparations of corresponding vascular walls, detection of periodontal pathogens was successful. It has been shown that microorganisms occurring in periodontitis are associated with arteriosclerosis (Mastragelopulos, et al., 2004). Periodontal pathogenic microorganisms lead to activation of platelet aggregation, promote the production of inflammatory molecules, as well as formation of foam cells from macrophages, leading to accumulation of atherosclerotic plaque in vessel walls (Jacek, 2018). Connective tissue of gingival margin has a dense, highly permeable subsulcular vascular network (Hellwege, 2018). When periodontium is destroyed, bacteria can enter the bloodstream via resulting gingival pockets, where they can endanger the systemic health of patients (Jacek, 2018). But it is not only via gingiva that microorganisms can spread throughout body. Silent aspiration, which is associated with an increased risk of pneumonia, as well as cracked mucous membranes are considered other possible routes of spread (Elsäßer & Ludwig, 2017). It becomes clear that oral infections can be the starting point for development of further diseases (Hellwege, 2018). In addition to already described interaction with atherosclerosis, links to diseases endocarditis, coronary heart disease and stroke, among others, are assumed. A correlation with insulin requirements of diabetics and complications in pregnancy is also suspected (Hellwege, 2018). It should be noted that with help of better dental health, general health can benefit. The quote by Klaus-Dieter Hellwege from the book *“Praxis der zahnmedizinischen Prophylaxe”* (The Practice of Dental Prophylaxis), published by *Thieme Verlag* in 2018, concluded: "With clean and healthy teeth, life is not only better, but also longer." (Quote, Klaus-Dieter Hellwege, 2018, Chapter 3, p.47). Furthermore, good oral hygiene makes a positive and decisive contribution to well-being and quality of life (Elsäßer & Ludwig, 2017).

Based on the above considerations, the following hypothesis can be formulated: If good oral hygiene can no longer be achieved due to impeded oral care, this leads to patient discomfort. Aided by the inhibited possibilities of oral care, food intake and fluid intake, the affected patients distance themselves from their social environment (Jakobsen & Sticher, 2015).

In summary, on the one hand it becomes clear that good oral hygiene and oral health have great relevance for a high quality of life, participation in everyday life and a healthy systemic and psychological set-up. On the other hand, it can be stated that a deterioration of oral hygiene has far-reaching consequences for health and such a situation should be recognized and treated promptly. Especially in predilection sites such as fissures, active plaque can be deposited and, if removed irregularly and delayed, can lead to demineralisation on tooth surface. Resulting caries lesions are irreversible (Roulet, Fath, & Zimmer, 2017).

Based on above-mentioned indications that oral hygiene and associated oral health could be impaired in patients with facial nerve palsy due to the fact that oral care is more difficult and that self-cleaning mechanisms are restricted, an overview of oral health of patients with facial nerve palsy at Jena University Hospital is to be created. If possible deficits and differences to non-affected persons can be uncovered, it will be possible to derive a need for dental therapy, to specifically address this patient group in the future and to improve their oral hygiene and associated general health.

# 3 Objectives

## 3.1 Primary objective

The primary aim of the study is to examine and record oral health of patients with different forms of facial nerve palsy and document their oral health in relation to facial nerve palsy variables such as duration and cause of disease, which are collected as part of routine investigation of the Facial Nerve Centre Jena. Special attention will be paid to periodontal health of patients. In order to record oral health, a complete dental status including existing restorations and tooth mobility should be collected. To assess periodontal and gingival health, PSI (parameter for chronic periodontal disease, developed by the German Society of Periodontology 2002, cf. Hellwege 2018), presence of calculus, maximum probing depth (in mm), attachment loss (in mm), mod. SBI according to Lange et al. 1986 (parameter for occurrence of sulcus haemorrhage, cf. Weber 2017) as well as PBI according to Saxer and Mühlemann 1975 (parameter for determining the quantity of papillary haemorrhage, cf. Hellwege 2018) should be determined by a dentist. As further oral health parameters, caries and plaque infestation should be documented. Caries infestation is to be determined using PUFA index according to Monse et al. 2010 (parameter for odontogenic infections due to untreated caries, cf. Kühnisch & Heinrich-Weltzien, 2020) and recording of caries activity. Plaque infestation is examined with TI (Turesky et al. 1970, parameter for plaque infestation, modification of plaque index according to Quigley and Hein 1962, cf. Peter Gängler et. al 2005) and API according to Lange et al. 1986 (parameter for approximal/interdental plaque infestation, cf. Weber 2017). In addition, oral mucosa should be inspected thoroughly and presence of halitosis assessed using organoleptic diagnostics (cf. Weber 2010).

This will provide a comprehensive overview of oral situation in patients with facial nerve palsy. Individual oral health parameters will be compared with a control group, which will be examined with regard to same measurement procedures.

## 3.2 Secondary objective

Secondary objective is to determine oral health-related quality of life of patients with the validated OHIP-G14 questionnaire, in order to be able to address the psycho-social component of disease. Results of questionnaires are compared with variables of facial nerve palsy, such as duration and cause of disease, which are collected as part of routine examination of Jena Facial Nerve Centre, as well as with values of a control group.

# 4. Study design and description

## 4.1 Type of study

The planned study is a controlled cross-sectional study.

## 4.2 Type of therapy assignment

No therapies will be performed on patients in sense of a study-related intervention. Routine dental examinations will be performed as indicated.

If desired, patients can receive expert advice for optimal oral care from the dentist during or after the examination and clarify open questions.

## 4.3 Type of comparison group

A corresponding control group of volunteers not suffering from facial nerve palsy will be recruited in exactly the same way as the patient group from the setting of ambulance level and ward level of the ENT-clinic of Jena University Hospital.

In event that too few controls can be recruited from this setting for good comparability, study-related examinations are carried out on corresponding controls from Tinnitus Centre of the ENT- clinic of Jena University Hospital or on patients from dental practices established in Thuringia.

The study will be matched according to age and gender.

## 4.4 Scope of the study

A total of 60 patients and 60 controls will be included. The exact sample size was planned with the Institute for Medical Statistics, Informatics and Data Science Jena using the programme G*power.

## 4.5 Patient and subject recruitment

Both collectives will be recruited and examined by the same examiner.

Patients are recruited from the ENT-clinic of Jena University Hospital during EMG consultation hours on Tuesdays, within the framework of part-time inpatient facial nerve palsy training of Jena Facial Nerve Centre and patients with acute facial nerve palsy within the inpatient stay.

Recruitment of suitable controls also takes place in the ENT-clinic of Jena University Hospital. Controls will be included who are undergoing inpatient treatment but not due to facial nerve palsy. Suitable controls must also not have suffered facial nerve palsy in their past. Furthermore, an attempt will be made to recruit patients via the Tinnitus Centre at Jena University Hospital. Furthermore, patients can be recruited from dental practices established in Thuringia, if this is necessary for a better comparison result to the patient group.

## 4.6 Schedule

Expected total study duration: 44 months

Milestones (month/year):

| Preparations/planning | 05/2020 |
| --- | --- |
| Inclusion of first patient | 08/2020 |
| Inclusion of last patient | 05/2021 |
| Inclusion of first volunteer | 08/2020 |
| Inclusion of last patient | 07/2021 |
| End of statistical analysis | 12/2022 |
| Final report | 12/2023 |

# 5 Selection

## 5.1 Inclusion criteria

**Patients n=60**

- facial nerve palsy diagnosed by a doctor
- all ages
- written informed consent from patient or legal guardian (for patients under 18 years of age) to participate in study
- toothed
- not suffering from any of following diagnosed diseases: Haemophilia A and B, Epilepsy, Aids

**Controls n=60**

- selection with regard to age and sex as a control group to previously examined patients
- no facial nerve palsy ever diagnosed
- written consent of the subject or legal guardian (for subjects under 18 years of age) to participate in the study
- toothed
- not suffering from any of the following diagnosed diseases: Haemophilia A and B, Epilepsy, Aids

## 5.2 Exclusion criteria

**Patients**

- multimorbid patients
- no diagnosed facial nerve palsy
- lack of consent from patient or legal guardian (for patients under 18 years of age) to participate in study
- edentulous
- suffering from one of following diagnosed diseases: Haemophilia A and B, Epilepsy, Aids

**Controls**

- multimorbid subjects
- age and gender mismatch with previously studied patients
- diagnosed facial nerve palsy currently or in past
- lack of consent from control or legal guardian (for subjects under 18 years of age) to participate in study
- edentulous
- suffering from one of following diagnosed diseases: Haemophilia A and B, Epilepsy, Aids

# 6 Procedure of the study

## 6.1 Information and consent

At beginning of study, persons (from patient or control group) will be given an information sheet (see appendix), which provides information about nature of the study, objectives, procedure, duration, benefits, possible risks, data evaluation, data protection and participant rights, as well as a consent form (see appendix). After reading both forms thoroughly, the examiner ensures that the patient/control person has understood the information and gives him/her the opportunity to address any open questions and have them answered in detail. Persons have sufficient time to decide whether to participate in the study. In next step, participant will be asked, if he or she is interested in the study, to document his or her knowledge and consent in writing. This consent also explicitly refers to collection and processing of personal data. Based on this, patients/controls will be informed in detail and explicitly about purpose and scope of collection and use of this data.

Patients/controls will subsequently receive a copy of signed consent form, original will remain with the examiner. Participants can withdraw this consent at any time without giving reasons and without detriment to his/her further treatment, discontinue the study and withdraw from the study. In this case, time of discontinuation of participation will be documented. If the consent form is not signed, subject will not be included in study.

Only when the person has affirmatively signed both forms, the person has been given an appropriate copy of documents and all other questions have been clarified, study will begin.

## 6.2 Questionnaire

Prior to examination of persons, a questionnaire (Questionnaire 1, subjective assessment of own oral hygiene and oral health, see Appendix) will be handed out to participants and discussed with the patient/control person in interview. In case patient/control person prefers to complete questionnaire alone, he/she will be allowed to do so. Patients/controls are asked to complete questionnaire as truthfully and intuitively as possible.

Questionnaire collects important information such as person's attitude towards his/her own oral hygiene, previous performance of dental cleaning as well as possible risk factors for development of poorer oral hygiene in order to determine whether occurring deficits in oral hygiene already existed before facial nerve palsy or whether the person may also have genetic or motor-related limitations, whereby poorer oral hygiene could no longer be directly attributed to facial nerve palsy. Questionnaire should therefore provide the examiner the opportunity to get to know the patient/control person better with regard to his/her oral hygiene and to be able to assess him/her more successfully with regard to this.

## 6.3 Oral examination

Oral examination of patients follows the same procedure as that of controls.

During oral examination, the examiner inspects the well-lit oral cavity and diagnoses teeth, gingiva and oral mucosa as well as existing dentures as oral structures. A complete dental status of the person including tooth restorations and their tooth mobility, periodontal health by means of PSI, presence of calculus, MPD as well as attachment loss, gingival bleeding by means of mod. SBI according to Lange et. al 1986 and PBI according to Mühlemann and Saxer 1975, caries infestation using PUFA index according to Monse et al. 2010 and caries activity, plaque infestation using API according to Lange et al. 1986 and TI according to Turesky et al. 1970, abnormalities in oral mucosa according to WHO classification and presence of halitosis were examined and documented. A paper-based report form (see appendix) is used to record and document the dental status and various oral health parameters, which is converted into an Excel document later. After findings have been recorded, intraoral photographs are taken.

If desired, patients can ask the examiner open questions regarding oral care and receive expert advice for optimal tooth cleaning and plaque management.

Examinations are carried out on premises of ENT department at Jena University Hospital. An examination of controls is also conceivable in a dental practice. Participating patients and controls will be examined and treated in a standardized manner in the ENT-clinic of Jena University Hospital with regard to their illness independently of the planned study. Data obtained on oral health of patients/controls will be communicated to them and treated confidentially, but will have no influence on further dental and ENT treatment of patients.

## 6.4 OHIP-G14 questionnaire

After oral examination, the person is given another questionnaire to reflect the perceived oral health-related quality of life. Therefore the validated OHIP-G14 questionnaire is used. The person is asked to fill this out truthfully and intuitively as well. Alternatively, questionnaire will be discussed in interview.

## 6.5 Documentation

Pseudonymous data collection will be carried out with a patient/control identification list (see appendix), a questionnaire to be filled in manually (see appendix) and two questionnaires to be filled in manually (see appendix). Paper-based questionnaire will be converted into a digital Excel form.

## 6.6 Data analysis

Data will be analysed with regard to individual oral health parameters for each patient/control person. Parameters are also evaluated separately for paretic and non-paretic side of patients mouths and for left and right side of controls in order to be able to make a side-by-side comparison.

With data from questionnaires, an evaluation is carried out for each question asked individually.

In addition, data is evaluated according to age, gender, form, duration and cause of facial nerve palsy.

## 6.7 Comparison of results

Data are first compared between patients and secondary with those of controls.

Comparisons are made with regard to oral health parameters:

- per patient:

- paretic and non-paretic side of mouth

- per controls:

- right and left side of mouth

- between patients:

- both halves of total mouth

- paretic side of mouth

- non paretic side of mouth

- between patients and controls:

- both sides of total mouth

- paretic side of mouth with corresponding side of mouth of control person

- non-paretic side of mouth with corresponding side of mouth of control person

- compared in terms of data from questionnaires:

- answers to each question between patients

- answers to each question between controls

- answers to each question between patients and controls

## 6.8 Tabular schedule

| 1. | information about the study and obtaining informed consent |
| --- | --- |
| 2. | handing out, answering and documentation of the 1st questionnaire  (Questionnaire 1, subjective assessment of own oral hygiene and oral health) |
| 3. | data collection during dental oral examination |
| 4. | documentation of data |
| 5. | handing out, answering and documentation of the 2nd questionnaire  (OHIP-G14 questionnaire on oral health-related quality of life) |
| 6. | analysis and evaluation of data |
| 7. | comparison of results |
| 8. | publication of results |

# 7 Biometrics and sample size planning

A similar study demonstrating that peripheral facial nerve palsy impairs food intake and worsens oral hygiene, which may lead to oral diseases, was conducted at Nabon University Itabashi Hospital and Nihon University Dental Hospital in Japan from 2009 to 2011. This study included 30 patients and 30 controls. The planned study is based on this.

The necessary number of participans was determined with the Institute for Medical Statistics, Informatics and Data Science Jena. The programme G*power was used. Following values were assumed when calculating the exact number of cases:

- Wilcoxon-Mann-Whitney test (two groups)
- Tail: two
- parent distribution: min ARE
- effect size d: 0.6666667
- α err prop: 0.05
- Power (1-β err prop): 0.8
- allocation ratio N1/N2: 1

Thus, a case number of 43 patients and 43 controls was calculated. This makes a total number of 86 participants. To take drop-out cases into account, a total number of 120 subjects (60 patients/controls per group) is assumed.

# 8 Data management

## 8.1 Identification list

Patients'/controls' data will be recorded pseudonymously. An individual pseudonym is used for each patient/control person, which alone does not allow to recognised identity of participants. A patient/control person identification list is kept. In this list, patient/control person identification number is linked to participant's full name, date of birth, age, occupation and, in case of patients, to form, severity, duration and cause of facial nerve palsy in order to enable subsequent identification of participants. This document will be kept absolutely confidential and will not come to third parties. It will be archived for 10 years.

## 8.2 Data collection/documentation forms

Data collection necessary for the study will take place in the ENT-clinic of Jena University Hospital and, if necessary, in established dental practices in Thuringia. Data will primarily be collected in form of paper-based documentation forms and transferred to corresponding digital Excel spreadsheets.

## 8.3 Storage of study documents

All study-related documents will be kept in original form with the examiner for 10 years after study completion. Documents will be kept confidentially in a secure location. Care will be taken to ensure that documentation records are not stored with patient/control person identification list.

# 9 Ethical considerations

Principles of "Declaration of Helsinki" (as of October 2013) are taken into account in planning of the study. No negative effects on patients and controls are to be expected as a result of the study. There is no risk for patients and controls. Treatment of patients will not be changed by the study. For participants, there is a one-time examination of 60 minutes due to the study.

Recommendations of "Good Clinical Practice", valid since 17.1.1997, will be taken into account, if applicable.

# 10 Funding

There is no compensation for participation. The study will be funded from budgetary resources. There are no other sponsors or conflicts of interest.

# 11 References

Elferich, B. & Tittmann, D. (2004). *Die Therapie des Facio-Oralen Ttrakts: F.O.T.T. nach kay Coombes.* (R. Nusser-Müller-Busch, Hrsg.) Berlin; Heidelberg; New York: Springer. S.78-79

Elsäßer, G. & Ludwig, E. (2017). Handbuch der Mundhygiene. In Bundeszahnärztekammer (Hrsg.). Berlin.

Gängler, P., Hoffmann, T., Schwenzer, N. & Willershausen, B. (2005)

*Konservierende Zahnheilkunde und Parodontologie.* Michael Ehrenfeld(Hrsg.), Zweite Aufl.

Stuttgart: Georg-Thieme-Verlag KG, S. 252

Hellwege, K.-D. (2018). *Die Praxis der Zahnmedizinischen Prophylaxe, Ein Leitfaden für die Individualprophylaxe für Zahnärzte und Mitarbeiter.* Stuttgart: Georg Thieme verlag KG.S. 35, S.46-47, S.88-90

Jacek, R. (November 2018). Parodontitis ist ein Risiko für den gesamten Körper. *ZMK aktuell* , S. 788-790.

Jakobsen, D. & Sticher, H. (2015). Die Therapie des Facio-Oralen Trakts: F.O.T.T. nach Kay Coombes. In R. Nusser-Müller-Busch (Hrsg.). Berlin, Deutschland: Springer.S.160-161,S.168, S.171

John, M. T. (2002). The German version of the Oral Health Impact Profile – Translation and psychometric properties. *European Journal of Oral Science* , S. 110,425-433.

Kaschke, O., Behrbohm, H. & Nawka, T. (2009). *Kurzlehrbuch Hals-Nasen-Ohren-Heilkunde.* Stuttgart: Georg Thieme Verlag KG.S.67

Kato, Y., Kamo, H., Kobayashi, A., Abe, S., Okada-Ogawa, A., Noma, N. et al. (Juni 2013). Quantitative evaluation of Oral Function in Acute and Recovery Phase of Idiopathic Facial Palsy; A Preliminary Controlles Study. *Clinical Otolaryngology* , S. 231-236.

Kühnisch, J. & Heinrich-Weltzien, R. (2020). *Kinderzahnmedizin.* (J. Kühnisch, Hrsg.) Berlin, Deutschland: Quintessenz-Verlags-GmbH.

Mastragelopulos, N., Rogge, S., Kielbassa, A., Haraszthy, V., Zambon, J., Brunkwall, J. et al. (April 2004). Parodontitis und Arteriosklerose. *Gefässchirurgie* .

Roulet, J.-F., Fath, S. & Zimmer, S. (. (2017). *Zahnmedizinische Prophylaxe: Lehrbuch und Praxisleitfaden.* Deutschland: Elsevier, Urban&Fischer.S.58, S.60, S.68

Slade, G. D. (1997). Derivation and validation of a short‐form oral health impact profile. *Community Dentistry and Oral Epidemiology* , S. 25(4), 284-290.

Trepel, M. (2017). *Neuroanatomie, Struktur und Funktion.* Deutschland: Elsevier, Urban&Fischer.S.68, S.122, S.148

Weber, T. (2010). *Memorix Zahnmedizin.* Stuttgart, New York: Georg-Thieme-Verlag KG. S.109

Weber, T. (2017). *Memorix Zahnmedizin*. Stuttgart, New York: Georg-Thieme-Verlag KG. S.118-126

Zenk, J., Leins, P. & Bozzato, A. (2005). *HNO Praxis heute, Funktionsstörungen und funktionelle Störungen.* (E. Biesinger, & H. Iro, Hrsg.) Heidelberg: Springer. S.2

# 12 Attachments

Patient group information sheet

Information sheet control group

Informed consent form patients

Informed consent form test persons

Identification list

Questionnaire 1

OHIP-G14 questionnaire

Findings sheet
