# Supplementary material for: Oral health and oral health-related quality of life in patients with chronic peripheral facial nerve palsy with synkineses—A case-control-study
Source: PLoS One. 2022 Nov 17;17(11):e0276152. doi: 10.1371/journal.pone.0276152 (PMC9671450; doi:10.1371/journal.pone.0276152)
Supplement: Attachment — Submitted filename: Remarks Plos One.pdf [file pone.0276152.s008.pdf]


General remarks
- This paper has been well elaborated, no doubt. Notwithstanding, the Authors have missed to strictly follow the Journal style, see Guidelines, and consult some recently published Plos One papers.
- Do not submit two-column format.
- This revised and re-submitted draft would benefit from language editing. Please search some help from a native speaker, to polish your text, and to facilitate reading. Remember that Plos One does not provide an in-depth editing service.

Abstract
- Double check and revise for correct sub-headlines. For example, Plos One style is "Materials and methods".
- "It was also observed that the paretic side compared to non-paretic side of patients was significantly more affected by inflammatory periodontal diseases and had a higher prevalence of caries." What means "significantly more"? Please add exact p values.
- Same with "Patients with FP described a greater limitation of the OHQoL than patients without this disease." What means "greater"? Plos One style would be "(p = 0.838", or "(p < 0.001)", please revise thoroughly.
- "Patients with synkineses after acute FP suffer from significantly more dental caries, periodontal disease and impairment of OHQoL compared to people without FP." This is a simple repetition of your results.
- "Intensive preventive dental care and specific oral hygiene training to prevent a double burden of disease are strongly recommendable." This might be right, but this does not refer to your aims.
- Remember that your conclusions should be appropriately stated, must be connected to the original question investigated, and have to be limited to those supported by the results.
- With your revision, please stick to the Journal's maximum word limit. Please please provide as much information as possible within the allowed 300-word limit.

Intro
- Please consult some recently published Plos One papers. No numbering of headlines mandatory. "1 Introduction" must read "Introduction".
- References must be according to Journal style. "Facial nerve palsy (FP) is the most common cranial nerve lesion (1)." must read "Facial nerve palsy (FP) is the most common cranial nerve lesion [1]." Revise thoroughly.
- Divide your text into digestible units, please. Currently, this section represents ONE single paragraph.
- "A study [6] gives an initial indication (...)." What kind of study do you refer to here? A previous/recent one? A study focussing on what? This study has been finished and published already, right? So switch to present perfect, please.
- "Furthermore, it has not yet been clarified whether handedness of affected patients has an influence in this regard." Please compare to your Abstract section, and revise to cover this aspect.
- With reference to "1.1 Aims of the study", see comments given above. No subheading necessary here. Remember that with the current section, your only task is to elaborate both aims and objectives. Thus, no subheadings like "1.1 (...)" mandatory.
- Instead of working hypothesis, please provide sound and valid null hypothesis.

Meths
- Again, with "2 Material and methods", see comments given above, and revise thoroughly.
- "To detect an effect size of Cohen's d=0.67 with a power of 80% in total 86 patients (43 per group) need to be analysed (G*Power 3.1, two sided Wilcoxon-Mann-Whitney Test, significance level 5%; Figure 1)." Why did you stick to an "effect size of Cohen's d=0.67", and to a "power of 80%"? Please provide sound references, and provide a convincing rationale.
- Please provide ethical vote number and date of approval.
- "According to Landis/Koch, this corresponds to a strong agreement." First, this is an explanation, not a methodology. Stick to "Materials and methods here". Second, a reference would seem missing with your Landis/Koch explanation, which should be copied and pasted to the Discussion section.
- Same with "With regard to the Turesky Index, a substantial agreement of 0.75 was found between the two examiners." This clearly is a result.
- "The examiner, a calibrated dentist, used a blunt probe and a mirror." First, provide initials (in parentheses) of the "examiner". Second, with ALL materials (including chemicals) and methodologies (including statistical software), please use general names with your text, followed by (brand name; manufacturer, city, ST[ate, abbreviated, if US], country) in parentheses. Stick to semicolon. Revise thoroughly.
- "The handedness was determined with the Edinburgh Handiness Inventory (EHI)[15] questionnaire." See comments given above. You surely will agree that all aims must be reported, and all aims would call for results. Compare Abstract and Intro sections.
- Do not use legale term with your text. Delete "®", "Corp.", and so on.
- With regard to "IBM SPSS Statistics (Version 27)", see comments given above.

Results
- Please note that "infestation" would seem an unusual term. Please reduce, and polish your text.
- Your clinical Figures would seem perfectible, please revise, and add new ones. First, from a dental point of view, these photographs would not seem mandatory. Second, those Figures should only be followed if "representativeness" is ensured (please expand your legends).
- " What kinds of "electric toothbrushes" have been used?
- Regarding "(p<0.01)", "(p≤0.001)", or "(p=0.01)" (and so on), please see comments given above.

Disc
- "(...) was examined for the first time in such a large group and using such a detailed and standardized examination protocol." Don't be too proud of your work. This should be decided by the future readers, so please tone down.
- "The patient's handedness might explain these differences. (...)" Again, see comments given above, and revise carefully.
- "This study reports new findings on oral health in patients with FP." Again, see comments given above, and remember that "each and every study reports new findings (on the respective topic)".
- Remember that with your revision, you revised null hypothesis must be referred to. Note that H0 can be rejected or not rejected.
- "All patients were examined under the same conditions and according to the same procedure from the same calibrated examiner." This should be a strength? To some

extent, I would agree, but this is not convincing when reflecting on your limitation: "Due to the limited lighting and patients' position on a chair, oral health parameters might have been underestimated." Don't you think that this would seem controversial? Please revise carefully.
- "However, this was not possible due to restrictions caused by the Covid-19 pandemic." This would seem a bad excuse only.

Concl

- With your Conclusions, please stick exclusively to your revised aims. Do not simply repeat your results here. Do not speculate. Do not explain. Instead, provide a reasonable and generalizable extension of your outcome. Revise thoroughly.

Refs
- Please revise for uniform formatting.
- Stick to Journal style.
- "Style would be "Kasraei S, Sami L, Hendi S, Alikhani MY, Rezaei-Soufi L, Khamverdi Z. Antibacterial properties of composite resins incorporating silver and zinc oxide nanoparticles on Streptococcus mutans and Lactobacillus. Restor Dent Endod. 2014; 39(2): 109–114. https://doi.org/10.5395/rde.2014.39.2.109 PMID: 24790923" Provide doi and PMID numbers.

In total, this revised and re-submited draft would seem interesting, is considered easily intelligible, and should be worth following after some further revisions. Currently, it is not ready to proceed.
