# Supplementary material for: Oral health and oral health-related quality of life in patients with chronic peripheral facial nerve palsy with synkineses—A case-control-study
Source: PLoS One. 2022 Nov 17;17(11):e0276152. doi: 10.1371/journal.pone.0276152 (PMC9671450; doi:10.1371/journal.pone.0276152)
Supplement: Attachment — Submitted filename: Response to Reviewers.pdf [file pone.0276152.s009.pdf]

Prof. Dr. med. dent. Dr. h. c. Andrej M. Kielbassa
Academic Editor
PLOS ONE

Rebuttal to the reviewer

Manuscript: *"Oral health and oral health-related quality of life in patients with chronic peripheral facial nerve palsy with synkineses - a case-control-study"*

Ref.: PONE-D-21-21348

Dear Prof. Dr. Dr. Kielbassa,

We greatly appreciate the comments and the reviews of our manuscript submitted for publication in the prestigious journal *Plos One*. We are pleased to note that you feel that the paper is interesting and relevant in its field. The comments were helpful in identifying areas for improvement, clarification or addition of further details.

In this letter we provide point-by-point responses the reviewers' comments and explain all text and table changes made to the manuscript.

We look forward to your favorable review and remain at your disposal should any further information be required.

With many thanks on behalf of all co-authors,

Yours sincerely,

Lisa Strobelt

1. **Response to General remarks**

1.1 Concern of the Reviewer

This paper has been well elaborated, no doubt. Notwithstanding, the Authors have missed to strictly follow the Journal style, see Guidelines, and consult some recently published Plos One papers.

Our response

Thank you very much for the appreciative feedback. During the revision process, we have taken a closer look at the submission guidelines of the journal and studied previously published articles to meet the publication criteria.

1.2 Concern of the Reviewer

Do not submit two-column format.

Our response

Thank you very much for this advice. We have changed the format to one-column-format.

1.3 Concern of the Reviewer

This revised and re-submitted draft would benefit from language editing. Please search some help from a native speaker, to polish your text, and to facilitate reading. Remember that Plos One does not provide an in-depth editing service.

Our response

Thank you very much for this advice. We had the manuscript checked again by an English teacher and native speakers and hope very much that the language and form of expression could be optimized as a result. To value their help, we added the chapter *"Acknowledgments"*.

Revised text (line 717-719)

Acknowledgments

We thank C. Kaden for his technical support and for helping us with Excel. Additionally, we thank X. Müller, M. Bober-Reeves and T. Bober for correcting the English language and expression.

2. **Response to Abstract**

2.1 Concern of the Reviewer

Double check and revise for correct sub-headlines. For example, Plos One style is "Materials and methods".

Our response

Thank you very much for this advice. We corrected the sub-headline from "Material and methods" to "Materials and methods" (line 130). In the abstract, we removed the subheadings altogether and created a coherent text, as this was the case for the majority of articles published in PLos One such as the article on *Biomass residues improve soil chemical and biological properties reestablishing native species in an exposed subsoil in Brazilian Cerrado* from 27.06.22 or the article on *Climate policy support as a tool to control others' (but not own) environmental behaviour?* from 22.06.22. Among the recently published articles, we could only see subheadings in the article *on HBV seroprevalence and liver fibrosis status among population born before national immunization in Southern Thailand: Findings from a health check-up program* from 24.06.22. However, these were again structured differently in *background, (just) methods, results and conclusion*, so that we decided to reduce the subheadings altogether and to orient ourselves to the other articles. We also removed the sentence *Jena University Hospital*

Ethics Committee approved this study (Reg. 2020-1883-BO). from the abstract, because in the other published articles no information is given in regard of this in the abstract, but it is referred to later in the material and methods section (line 134/135).

Revised text (line 130/131)

Materials and methods

Recruitment and Inclusion and Exclusion Criteria

2.2 Concern of the Reviewer

"It was also observed that the paretic side compared to non-paretic side of patients was significantly more affected by inflammatory periodontal diseases and had a higher prevalence of caries." What means "significantly more"? Please add exact p values.

Our response

Thank you very much for this suggestion for improvement. We specified the statement and added the corresponding p-value. We also added appropriate spaces before and after the mathematical symbols.

Revised text (line 51-53)

The paretic side compared to non-paretic side of patients was significantly more affected by inflammatory periodontal diseases ($p = 0.032$) and had a higher prevalence of caries ($p = 0.163$).

2.3 Concern of the Reviewer

Same with "Patients with FP described a greater limitation of the OHQoL than patients without this disease." What means "greater"? Plos One style would be "(p = 0.838", or "(p < 0.001)", please revise thoroughly.

Our response

Thank you very much for this suggestion for improvement. We specified the statement and added the corresponding exact p-value. We also added appropriate spaces before and after the mathematical symbols.

Revised text (line 55-56)

Patients with FP described a greater limitation of the OHQoL than patients without this disease ($p < 0.001$).

2.4 Concern of the Reviewer

"Patients with synkineses after acute FP suffer from significantly more dental caries, periodontal disease and impairment of OHQoL compared to people without FP." This is a simple repetition of your results.

Our response

Thank you very much for this assessment. We have removed this sentence from the abstract summary.

2.5 Concern of the Reviewer

"Intensive preventive dental care and specific oral hygiene training to prevent a double burden of disease are strongly recommendable." This might be right, but this does not refer to your aims.

Our response

Thank you very much for your assessment. We have rewritten the summary of the abstract and tried to create a more concrete connection to our aims.

Revised text (line 59-62)
In conclusion, facial nerve palsy affects oral health, oral hygiene and OHQoL of patients while handedness influences oral hygiene and oral health.

2.6 Concern of the Reviewer
Remember that your conclusions should be appropriately stated, must be connected to the original question investigated, and have to be limited to those supported by the results.

Our response
Thank you very much for your assessment. We have rewritten the summary of the abstract and tried to create a more concrete connection to our aims.

Revised text (line 59-62)
The conclusion of our research is that facial nerve palsy affects the oral health, oral hygiene and OHQoL of patients. Handedness influences oral hygiene and oral health of our patients.

2.7 Concern of the Reviewer
With your revision, please stick to the Journal's maximum word limit. Please please provide as much information as possible within the allowed 300-word limit.

Our response
Thank you very much for this advice. We shortened the abstract to 295 words.

3. Response to Introduction

3.1 Concern of the Reviewer
Please consult some recently published Plos One papers. No numbering of headlines mandatory. "1 Introduction" must read "Introduction".

Our response
Thank you for pointing out the correct formatting. We looked at other articles published in Plos One and have removed the numbering from all headings.

Revised text (line 65)
Introduction

Facial nerve palsy (FP) is the most common cranial nerve lesion [1]. It can be assumed an incidence of ….

3.2 Concern of the Reviewer
References must be according to Journal style. "Facial nerve palsy (FP) is the most common cranial nerve lesion (1)." must read "Facial nerve palsy (FP) is the most common cranial nerve lesion [1]." Revise thoroughly.

Our response
Thank you very much for your correction. We have improved all our references throughout the manuscript according to Journal style.

Revised text

Facial nerve palsy (FP) is the most common cranial nerve lesion [1]. (for example line 66)

3.3 Concern of the Reviewer
Divide your text into digestible units, please. Currently, this section represents ONE single paragraph.

Our response
We thank you very much for your suggestion for improvement and divided our introduction into several sections for a better readability.

3.4 Concern of the Reviewer
"A study [6] gives an initial indication (...)." What kind of study do you refer to here? A previous/recent one? A study focussing on what? This study has been finished and published already, right? So switch to present perfect, please.

Our response
Thank you for your feedback. We added more details to the study and changed the tense used.

Revised text (line 80-82)
A previous study [6], including a quantitative evaluation of oral function in acute and recovery phase of idiopathic facial palsy has gave an initial indication that peripheral facial paralysis impairs oral hygiene and can promote oral diseases.

3.5 Concern of the Reviewer
"Furthermore, it has not yet been clarified whether handedness of affected patients has an influence in this regard." Please compare to your Abstract section, and revise to cover this aspect.

Our response
Thank you very much for this advice. In order to point out directly in the abstract that we also wanted to take into account influencing factors such as handedness when evaluating the oral health and oral hygiene of the patients concerned, we included this aspect in the abstract.

Revised text (line 33)
It was the aim of this study to assess oral health, oral hygiene with the influence of handedness and oral health related quality of life in patients with chronic peripheral FP.

3.6 Concern of the Reviewer
With reference to "1.1 Aims of the study", see comments given above. No subheading necessary here. Remember that with the current section, your only task is to elaborate both aims and objectives. Thus, no subheadings like "1.1 (...)" mandatory.

Our response
We thank you for your comment and removed the subheading “1.1 Aims of the study”.

3.7 Concern of the Reviewer
Instead of working hypothesis, please provide sound and valid null hypothesis.

Our response
Thank you very much for this advice. Analogous to our four “working hypotheses” or alternative hypotheses, we added and provided four corresponding null hypotheses.

Revised text (line 100-116)

The following hypotheses were tested:

1. **H0:** Oral health and oral health related quality of life does not differ between patient with and without FP.

   Ha: Oral health and oral health related quality of life differs between patient with and without FP.

2. **H0:** In patients with FP, oral disease does not affect the PS more than the NPS.

   Ha: In patients with FP, oral disease affects the PS more than the NPS.

3. **H0:** Oral health is not influenced by the individual oral hygiene behavior of patients with FP.

   Ha: Oral health is influenced by the individual oral hygiene behavior of patients with FP.

4. **H0:** The oral health and oral hygiene behavior of patients with FP do not depend on patient-related factors such as the handedness, the duration and perception of the disease and the disease-related quality of life.

   Ha: The oral health and oral hygiene behavior of patients with FP depends on patient-related factors such as the handedness, the duration and perception of the disease and the disease-related quality of life.

4. Response to Materials and methods

4.1 Concern of the Reviewer

Again, with "2 Material and methods", see comments given above, and revise thoroughly.

Our response

Thank you very much for pointing this out again. We removed the numbering from all headings and corrected “Material and methods” to “Materials and methods”.

4.2 Concern of the Reviewer

*"To detect an effect size of Cohen’s d=0.67 with a power of 80% in total 86 patients (43 per group) need to be analysed (G*Power 3.1, two sided Wilcoxon-Mann-Whitney Test, significance level 5%; Figure 1)." Why did you stick to an "effect size of Cohen’s d=0.67", and to a "power of 80%"? Please provide sound references, and provide a convincing rationale.*

Our response

The sample-size planning targeted to prove a moderate effect of FP on oral health parameters, this moderate effect was presumed to have clinical relevance. Because we could not use any previous studies for orientation, we proceeded generically. A Cohen's d of 0.5 to 0.8 describes a moderately strong effect (*J. Cohen: Statistical Power Analysis for the Behavioral Sciences. 2. Auflage. Lawrence Erlbaum Associates, Hillsdale 1988, ISBN 0-8058-0283-5.).* In order to commit to a value within this range, we assumed a value lying approximately in the middle of this range.
With regard to the power of 80%, we oriented ourselves to the generally accepted standard value.

4.3 Concern of the Reviewer

Please provide ethical vote number and date of approval.

Our response

We thank you for this advice and added ethical vote number and date of approval as follows.

Revised text (line 134)

Jena University Hospital Ethics Committee approved this study (Reg. 2020-1883-BO) on 27/08/2020.

4.4 Concern of the Reviewer

"According to Landis/Koch, this corresponds to a strong agreement." First, this is an explanation, not a methodology. Stick to "Materials and methods here". Second, a reference would seem missing with your Landis/Koch explanation, which should be copied and pasted to the Discussion section.

Our response

Thank you very much for this advice. We removed the results of the examiner's calibration from the materials and methods. Therefore we deleted the sentences *Based on evaluation using kappa-statistic, the examiner achieved an agreement of 0.86 with respect to the DMFT. According to Landis/Koch, this corresponds to a strong agreement. With regard to the Turesky Index, a substantial agreement of 0.75 was found between the two examiners* and gave them later in the Discussion section.

Instead, we only added methodologically that we used the kappa-statistic for the comparison of the two examiners and evaluated the calculated value with the help of Landis and Koch. We added the corresponding reference for the kappa-statistic and Landis/Koch.

Revised text

In materials and methods (line 165/166):

To compare the two examiners, the kappa statistic [8] was applied and the calculated value was evaluated using Landis/Koch [9].

In References (line 741/745):

7. J. Cohen:*Statistical Power Analysis for the Behavioral Sciences.* 2. Auflage. Lawrence Erlbaum Associates, Hillsdale 1988,ISBN 0-8058-0283-5.Kappa-statistic

8. Landis JR, Koch GG. The measurement of observer agreement for categorical data. Biometrics. 1977;33(1):159-74. https://pubmed.ncbi.nlm.nih.gov/843571/. PMID: 843571

4.5 Concern of the Reviewer

Same with "With regard to the Turesky Index, a substantial agreement of 0.75 was found between the two examiners." This clearly is a result.

Our response

You are absolutely right. Thank you for this correction. We removed the sentence from the Materials and Methods and gave the values later in the text.

4.6 Concern of the Reviewer

"The examiner, a calibrated dentist, used a blunt probe and a mirror." First, provide initials (in parentheses) of the "examiner". Second, with ALL materials (including chemicals) and methodologies (including statistical software), please use general names with your text, followed by (brand name; manufacturer, city, ST[ate, abbreviated, if US],

country) in parentheses. Stick to semicolon. Revise thoroughly.

Our response

Thank you very much for your comments. We have added the initials of the examiner in parentheses and details of the instruments used. In addition, we added the note that Mira2Ton staining solution was used to visualise the plaque.

Revised text

The examiner (L.S.), a calibrated dentist, used a blunt probe (periodontal probe; piece 973/CP15; Manufacturer Article no: 973/CP15; Item no.: 19329; Carl Martin GmbH; Solingen; Germany) and a mirror (mouth mirror standard no. 4 hollow; Manufacturer Article no. 70878; Item no. 70878; OMNIDENT Dental-Handelsgesellschaft mbH; Rodgau/Nieder-Roden; Germany). (line 175/178)
The oral cavity was illuminated with a head lamp (FaroMED-Effner; 08-365-GR; 4000-6500 LUX; Berlin; Germany).(line 180)
MIRA2Ton staining solution (Hager & Werken GmbH & Co. KG; Duisburg; Germany) was used to stain the plaque. (line 185/186)

4.7 Concern of the Reviewer

"The handedness was determined with the Edinburgh Handiness Inventory (EHI)[15] questionnaire." See comments given above. You surely will agree that all aims must be reported, and all aims would call for results. Compare Abstract and Intro sections.

Our response

Thank you very much for this advice. We completely agree with you. In the abstract, in relation to this aspect, we completed the sentence *It was the aim of this study to assess oral health, oral hygiene and oral health related quality of life in patients with chronic peripheral FP* in *It was the aim of this study to assess oral health, oral hygiene with influencing factors such as handedness and oral health related quality of life in patients with chronic peripheral FP.* We also added the aspect of handedness in the introductory part. In order to show the influence of handedness more precisely, we carried out an additional multiple linear regression analysis. The correlation proved to be non-significant. We added this aspect to the discussion.

Revised text

In the abstract (line 32-34):

It was the aim of this study to assess oral health, oral hygiene with the influence of handedness and oral health related quality of life in patients with chronic peripheral FP.

In the introduction part (line 111-116):

H0: The oral health and oral hygiene behavior of patients with FP do not depend on patient-related factors such as the handedness, the duration and perception of the disease and the disease-related quality of life.

Ha: The oral health and oral hygiene behavior of patients with FP depends on patient-related factors such as the handedness, the duration and perception of the disease and the disease-related quality of life.

In the Discussion part (line 598-600):

However, within a multiple linear regression analysis, this correlation could not be proven to be significant ($p = 0.541$).

4.8 Concern of the Reviewer
Do not use legale term with your text. Delete "®", "Corp.", and so on.

Our response
Thank you for pointing this out. We removed the corresponding designations.

Revised text (line 205-206)
The paper-based examination sheets and questionnaires were digitalized and data recorded with Microsoft Excel (Version 2007) by the investigating dentist.

4.9 Concern of the Reviewer
With regard to "IBM SPSS Statistics (Version 27)", see comments given above.

Our response
We thank you for this suggestion for improvement. We added more detailed information on the statistical programme used and made it clearer that all statistical calculations were carried out with this programme. Regarding the exact name of the statistical programme, we obtained information from the official website *(https://www.ibm.com/support/pages/how-cite-ibm-spss-statistics-or-earlier-versions-spss)*. However, abbreviations such as *Corp.* were used in this official designation *(IBM Corp. Released 2020. IBM SPSS Statistics for Windows, Version 27.0. Armonk, NY: IBM Corp)*, which we removed due to your previous comment.

Revised text (line 217-219)
All statistical analyses were carried out using IBM SPSS Statistics (IBM Released 2020. IBM SPSS Statistics for Windows, Version 27.0. Armonk, NY) and evaluated by the investigating dentist (L.S.) and a statistician (T.L.)

5. Results

5.1 Concern of the Reviewer
Please note that "infestation" would seem an unusual term. Please reduce, and polish your text.

Our response
Thank you for this advice. We reduce the term *"infestation"* in the entire text.

5.2 Concern of the Reviewer
Your clinical Figures would seem perfectible, please revise, and add new ones. First, from a dental point of view, these photographs would not seem mandatory. Second, those Figures should only be followed if "representativeness" is ensured (please expand your legends).

Our response
Thank you for your comment regarding our clinical figures. We have removed the figures from the manuscript.

5.3 Concern of the Reviewer
What kinds of "electric toothbrushes" have been used?

Our response

Thank you for this question. They were both electric rotary oscillating toothbrushes and sonic toothbrushes. We added this information to the text.

Revised text (line 332-334)

Furthermore, patients reported that they switched from electrical (both rotary oscillating electric toothbrushes and sonic toothbrushes) to manual toothbrushes due to changes in sensitivity.

5.4 Concern of the Reviewer

Regarding "(p<0.01)", "(p≤0.001)", or "(p=0.01)" (and so on), please see comments given above.

Our response

Thank you for bringing this point to our attention again. We added the appropriate spaces in the entire manuscript. We also checked the exact specification of the p-values and reported all p-values with an accuracy of 3 decimal places.

6. Response to Discussion

6.1 Concern of the Reviewer

"(...) was examined for the first time in such a large group and using such a detailed and standardized examination protocol." Don't be too proud of your work. This should be decided by the future readers, so please tone down.

Our response

Thank you for this feedback. It was not our intention to rate our work too highly. We have removed the relevant sentence *(In this case-control-study, oral health, oral hygiene behavior and oral health related quality of life in patients with chronic peripheral facial nerve palsy compared to healthy controls was examined for the first time in such a large group and using such a detailed and standardized examination protocol.)* from the manuscript and added the following sentence.

Revised text (line 431-433)

In this case-control-study, oral health, oral hygiene behavior and oral health related quality of life in patients with chronic peripheral facial nerve palsy compared to healthy controls was examined.

6.2 Concern of the Reviewer

"The patient's handedness might explain these differences. (...)" Again, see comments given above, and revise carefully.

Our response

Many thanks for this advice. We added the influencing of handedness for oral health and oral hygiene in the abstract, introduction and objective of the study.

6.3 Concern of the Reviewer

"This study reports new findings on oral health in patients with FP." Again, see comments given above, and remember that "each and every study reports new findings (on the respective topic)".

Our response

Thank you for this assessment. We have reduced the sentence from the manuscript.

6.4 Concern of the Reviewer

Remember that with your revision, you revised null hypothesis must be referred to. Note that H0 can be rejected or not rejected.

Our response

Sincere thanks for this advice. We indicated in the discussion whether our null hypotheses could be rejected or not. For the evaluation of the fourth null hypothesis, we integrated a new section into the discussion.

Revised text

In summary, our first and second null hypotheses can be rejected as explained and instead the corresponding alternative hypotheses can be proven.(line 502-504) ….. The first null hypothesis can also be rejected with regard to oral health-related quality of life of the patients with FP compared to our controls. (line 507-508)

…….All in all, our third null hypothesis can also be rejected in conclusion. (line 566-567) ……

Factors influencing oral health and oral hygiene behavior

With regard to our fourth null hypothesis, we can summarize the following. Within the subgroup analysis of the present study, a greater difference in plaque contamination between the two sides of the mouth was documented in patients with right-sided FP than in the patients with left-sided FP (right-sided FP: 0.7 vs. 0.5; $p \leq 0.001$; left-sided FP: 0.5 vs. 0.4; $p > 0.05$). The patient's handedness explain these differences. While in patients with left-sided FP the paresis was on the side of the mouth easier to clean for right-handers, right-handed patients with right-sided FP were exposed to double stress [42]. This finding shall be considered for designing individualized tooth brushing training. With regard to the disease-related quality of life, it was found that the lower the FaCE score, the more plaque covered the tooth surfaces. Additionally, in patients with lower FDI scores more missing teeth were diagnosed. These relationships were significant with a p-value of $p = 0.01$. Thus, it can be said that the patient-related parameter of disease-related quality of life influences oral health, although not with respect to all parameters. With regard to the duration of the paresis, no significant relationships with oral health and oral hygiene behaviour were found. Thus, it is not possible to clearly determine within patients suffering from facial nerve palsy whether the risk of oral health impairments is more likely to affect patients with the disease for a shorter or longer period of time. Therefore, it is important to take prophylactic dental measures in all patients with facial nerve palsy. In summary, the fourth null hypothesis can only be partially rejected. The patient-related parameters of handedness and disease-related quality of life have a partial influence on the oral health of the patients, but no significant relationships could be demonstrated with regard to the duration of paresis. (line 591-620)

6.5 Concern of the Reviewer

"All patients were examined under the same conditions and according to the same procedure from the same calibrated examiner." This should be a strength? To some extent, I would agree, but this is not convincing when reflecting on your limitation: "Due to the limited lighting and patients' position on a chair, oral health parameters might have been underestimated." Don't you think that this would seem controversial? Please revise carefully.

Our response

Thank you for bringing this possible contradiction to our attention. We agree with you that one could misunderstand these two sentences in combination. However, we rather wanted to express that the dental examination would certainly have been easier and better to perform in a dental practice. We reworded the sentence accordingly.

Revised text (line 665-670)

Nevertheless, the study has some limitations. Patients were examined in the hospital ward and not in a dental clinic. This made the collection of oral health parameters more difficult. More convenient for future studies would be the ability to transfer patients from the hospital to a dental office for oral examination.

6.6 Concern of the Reviewer

"However, this was not possible due to restrictions caused by the Covid-19 pandemic."
This would seem a bad excuse only.

Our response

Thank you for your concern regarding this statement. At the time of conducting the study, it was unfortunately not possible for us with our resources to transfer patients from the hospital to a dental office for oral examination just to conduct our study. That we consider a limitation of the study. We deleted the reasoning that this was done because of the Covid 19 pandemic from the manuscript.

7. Response to Conclusion

7.1 Concern of the Reviewer

With your Conclusions, please stick exclusively to your revised aims. Do not simply repeat your results here. Do not speculate. Do not explain. Instead, provide a reasonable and generalizable extension of your outcome. Revise thoroughly.

Our response

Thank you very much for this comment. We carefully revised the summary and removed repetitions, explanations and speculations. We very much hope that the new summary is more accurate and meaningful.

Revised text (line 675-681)

Patients with FP have poorer oral health and oral hygiene, especially on the side of the face affected by FP, and poorer OHQoL than patients without FP. Individual oral hygiene behaviour as well as patient-related parameters such as handedness or disease-related quality of life influence the oral health of patients. Adding oral health care to the existing therapy guidelines [7] and establishing an interdisciplinary cooperation might prevent oral health from being neglected producing unpleasant consequences. We therefore recommend that patients be referred to a dentist in addition to the other disciplines once a diagnosis has been made.

8. Response to References

8.1 Concern of the Reviewer

Please revise for uniform formatting. Stick to Journal style."Style would be "Kasraei S, Sami L, Hendi S, Alikhani MY, Rezaei-Soufi L, Khamverdi Z.Antibacterial properties of composite resins incorporating silver and zinc oxide nanoparticles on Streptococcus mutans and Lactobacillus. Restor Dent Endod. 2014; 39(2): 109–114. https://doi.org/10.5395/rde.2014.39.2.109 PMID: 24790923" Provide doi and PMID numbers.

Our response

Thank you very much for your comment on the formatting of the references. We carefully and conscientiously revised all references and adapted them to the required Journal style. We added PMID numbers and doi.

Revised text

For example (line 721-724):

1. Zimmermann J, Jesse S, Kassubek J, Pinkhardt E, Ludolph AC. Differential diagnosis of Peripheral facial nerve palsy: a retrospective clinical, MRI and CSF-based study. J Neurol. 2019; 266(10):2488-2494. https://pubmed.ncbi.nlm.nih.gov/31250103/ PMID:31250103. doi:10.1007/s00415-019-09387-w

8.2 Concern of the Reviewer

In total, this revised and re-submited draft would seem interesting, is considered easily intelligible, and should be worth following after some further revisions. Currently, it is not ready to proceed.

Our response

Thank you very much for your appreciative and helpful feedback on our manuscript. We have tried to adapt all your advice during our revision and to optimize our manuscript as best as possible.
